# Cecal microbiome transplantation without antibiotic preconditioning standardizes murine microbiomes

**DOI:** 10.3389/fmicb.2025.1632210

**Published:** 2025-08-11

**Authors:** Rye Howard-Stone, Philip Gerwin, Darien Capunitan, Mark Driscoll, Eric Jackson, Thi Dong Binh Tran, Ion Măndoiu, Elias Oziolor

**Affiliations:** ^1^School of Computing, University of Connecticut, Storrs, CT, United States; ^2^Pfizer, Groton, CT, United States; ^3^Intus Biosciences, Farmington, CT, United States

**Keywords:** microbiome analysis, cecal microbiome transplant, amplicon sequencing, antibiotic resistance, mouse microbiota, ASV resolution, microbiome standardization

## Abstract

**Introduction:**

Translation of nonclinical findings from laboratory mice to the clinic may be confounded by un-controlled variance in bacterial gut content, as a driver of immune maturation and recruitment, as well as drug metabolism. Understanding and controlling for microbiome variation in animal experiments can lead to better reproducibility of animal findings, more translatable characterization of efficacy and toxicity end-points and time and cost savings associated with pharmaceutical development. Microbiome composition has been linked to failure of translation of drug responses.

**Methods:**

In an effort to test the stability of microbiome introduction, we compare various methods for establishing a well-characterized, stable bacterial community in laboratory mice via Cecal Microbiome Transplant (CMT) with and without antibiotic preconditioning.

**Results:**

We demonstrate a single CMT treatment protocol effectively treats outbred mouse populations with two different initial gut bacterial profiles, causing the populations to converge to a third, more wild-type bacterial genetic environment suitable for initiation of nonclinical studies. We show that ASV-based monitoring provides the highest resolution for identifying and tracking bacterial profile differences, which can be obscured at the species level. We find that antibiotic preconditioning reduces efficiency for uptake of CMT-specific strains. Instead, antibiotics introduce uncontrolled variance in the resulting microbiome composition.

**Conclusions:**

We propose that CMT without antibiotic preconditioning provides increased control over host microbial composition, enabling expanded utility, accuracy, and relevance for nonclinical drug toxicity and therapeutic effect studies in laboratory mice, with minimal additional costs.

## 1 Introduction

Nonclinical drug trials often include laboratory mice, a powerful model for exploration of candidate drug effects. Laboratory mice offer controlled genetics, are raised in controlled environments, are inexpensive, and readily available. Mouse studies provide insights into the safety and efficacy of potential drug candidates prior to human testing. Increasingly, breakthrough drugs targeting previously untreatable autoimmune and inflammatory diseases are being designed that recruit the body's natural immune system defenses. These drugs activate or modulate innate and/or adaptive immune responses that respond to infection, control tumor growth, and regulate immune response. Unfortunately, these treatments may unintentionally activate or suppress the immune system in unwanted ways. There is growing evidence that laboratory mouse immune response depends on bacterial/host interactions (Sharma et al., 2019; Beura et al., 2016; Sadrekarimi et al., 2022; Zheng et al., 2020). For decades, commercial rodent vendors and institutions have eradicated and excluded adventitious organisms in order to reduce infection-related variability and therefore improve reproducibility. While this has been a valuable approach to increase throughput of mouse availability, it inadvertently produced mice with reduced microbial diversity and understimulated, less mature immune systems (Ericsson and Franklin, 2021). For example, reports detail nonclinical mouse studies that have failed to identify acute, life-threatening drug reactions that can occur via activation of inflammatory T-cells and resulting cytokine storms (Rosshart et al., 2019). These insights indicate that careful design and control of the mouse model microbiome may be a path to more translatable nonclinical results.

In this study, we tested the stability, efficiency and reproducibility of introduction of a diverse mouse microbiome in a nonclinical vivarium setting. To test these effects, we set up control and experimental conditions, which included introduction of diverse microbiomes through cecal microbiome transplants (CMT), across two vivaria (Groton, CT and La Jolla, CA) with 20 CD1 outbred mice per condition. We tracked strain level microbial fecal contents, using 2,500 bp 16S-ITS-23S amplicon sequence variants (ASVs), because shorter commonly used 16S amplicons cannot match longer amplicon accuracy (Johnson et al., 2019; Graf et al., 2021). Rather than relying on taxonomic comparisons, ASV sequences themselves were directly compared at the start of the experiment and over the next 60 days to determine effects on CMT introduction, to compare population changes at the sites over time, and to determine the level of bacterial identification required for unambiguous tracking of bacterial microbial content. The goals of the study were:

- to measure the initial gut bacterial population differences between laboratory mice sourced from a single vendor;
- to identify the stability of microbial profiles over time and across vivaria; and
- to understand the efficacy and stability of introduction of a diverse “wild-type” microbiome from a commercial vendor.

We found significant initial microbiome variation in populations of the same outbred stock of mice, sourced from the same vendor. Similar microbiome variability across mice from identical sources has previously been observed in other contexts, along with measurable impacts on experimental outcomes (Mandal et al., 2020). Tracking of both populations revealed changes resulting from CMT shifted bacterial strain profiles over the course of a few weeks, and that monitoring at the species level, resolution achieved by commonly targeted 16S subregions like V4 (Johnson et al., 2019), can disguise significant changes that can be observed at the ASV/strain level. Direct ASV comparison revealed that database-dependent taxonomic assignment of species or strains can fail to identify microbial population replacement of closely related strains. Furthermore, antibiotic treatment prior to introduction of a new microbiome can prevent establishment of a consistent microbiome starting point across populations. We identified a method successfully used to transplant a well-characterized gut microbiome into both populations of mice with initially different microbiome keystone species, which may provide a stable starting point for nonclinical studies.

## 2 Materials and methods

### 2.1 Animals

Eighty female and 80 male 6–9 week old CD-1 [Crl:CD1(ICR)] mice (Charles River Laboratories; Hollister, CA; Raleigh, NC) were used for this study. This outbred stock has been used historically for general toxicology studies at Pfizer. Virus Antibody Free mice were ordered from Charles River with a specific pathogen-free (SPF) status for mouse hepatitis virus, mouse kidney parvovirus, mouse rotavirus, lymphocytic choriomeningitis virus, ectromelia virus, mouse parvovirus, minute virus of mice, murine norovirus, pneumonia virus of mice, reovirus type 3, Sendai virus, Theiler mouse encephalomyelitis virus, mouse adenovirus, K virus, polyoma virus, mouse cytomegalovirus, mouse thymic virus, Haantan virus, lactic dehydrogenase elevating virus, *Filbacterium rodentium, mycoplasma pulmonis, Bordatella bronchiseptica, Streptococcus pneumoniae, Pasteurella* spp., *Helicobacter* spp., *Salmonella* spp., *Streptobacillus moniliformis, Clostridium piliforme, Corynebacterium kutscheri, Citrobacter rodentium, Encephalatizozoon cuniculi*, and endoparasites and ectoparasites. All mice were singly housed in disposable solid-bottom polyethylene terephthalate cages (Innocage, Innovive, San Diego, CA) with an individually ventilated cage lid (MVX6, Innovive) on an individually ventilated cage rack (Innorack 3.5, Innovive) set at 70 air changes per hour with negative pressure to the room. Cages were prefilled with irradiated α-cellulose bedding (Alpha-Dri, Shepherd Specialty Papers, Watertown, TN) and animals were provided with irradiated nesting material (Bed-r'Nest, The Andersons Lab Bedding, Quakertown, PA). Certified γ-irradiated rodent chow (Teklad Global 16% Protein Rodent Diet 2916C, Teklad^TM^ Diets, Madison, WI) and chlorinated water (Aquavive, 1–3 ppm, Innovive) were provided ad libitum. Cages were handled in and changed every 14 days in a HEPA-filtered, class II type A2 biologic safety cabinet. The holding room was ventilated with 100% filtered outside air (pre-filtered, box-filtered, HEPA-filtered) at 12 air changes hourly. Other room conditions were maintained at a temperature of 72°F (range, 69–76°F), relative humidity at 50% (range, 35%–60%), and a 12:12-h light:dark photoperiod.

### 2.2 DNA extraction and sequencing

Fecal samples were processed and analyzed as previously described (Graf et al., 2021). Briefly, fecal DNA was purified, PCR amplified, and pooled for sequencing using the Shoreline Complete StrainID Kit [StrainID set A (barcodes 1–96); Intus Biosciences, formerly Shoreline Biome, Farmington, CT] according to the manufacturer's instructions. Amplicon libraries were created using the SMRTbell express template prep kit 2.0 (catalog number 100-938-900; PacBio). The library was sequenced on a Sequel IIe system (Pacific Biosciences) at GeneWiz/Azenta, South Plainfield, NJ, USA. Circular Consensus Sequencing (CCS) reads with exact barcode matches were demultiplexed and pooled as described in the Supplemental material for ASV inference using DADA2 version 1.24.0 (Callahan et al., 2019, 2016a), running with R version 4.2.1. SBanalyzer 2.4 (Intus Biosciences, formerly Shoreline Biome) was used to map ASVs to the Athena database and assign taxonomic identification. Note that taxonomies were assigned for the purpose of demonstrating how taxonomic labels can obscure important ASV shifts in bacterial communities. Larger databases may obscure fewer shifts.

### 2.3 Experimental design

This experiment was conducted in parallel at two sites: Groton CT, and La Jolla, CA. At each site, 10 animals per sex were placed into four treatment groups:

1. Untreated (UNTR)
2. Antibiotic 10 days (ABX10)
3. Antibiotic 3 days followed by CMT (ABX3CMT)
4. CMT

Beginning on day 1, ABX10 and ABX3CMT received 0.25 ml of antibiotic solution twice daily via oral gavage. Antibiotic solution consisted of 10 g/L each of Ampicillin, neomycin, and metronidazole; and 5 g/L of vancomycin resulting in approximate doses of 100 mg/kg of ampicillin, neomycin, and metronidazole; and 50 mg/kg vancomycin. CMT (Trubiome^®^, Taconic, Rensselaer, NY) was administered on day 4 for both CMT and ABX3CMT groups. A single lot of donor material was generated by pooling cecal contents from up to 14 donor mice, thoroughly homogenized and distributed across 20 aliquots. These were randomly assigned to treatment groups and evenly split between sites. While minor lot-to-lot variation is possible, our approach uses a master homogenate to control for this source of experimental variability. 0.2 ml of Trubiome^®^ feces were thawed and mixed at a 1:30 dilution in sterile phosphate buffered saline, and given once via oral gavage. Fecal samples were collected on days 0, 1, 3, 5, 8, 10, 30, and 60. Day –2 samples are from three days prior to day 1, the day after the mice arrived onsite. At each collection time point, each mouse was placed in a cage on a fresh sterile drape. Gloves were disinfected with 70% ethanol between animals within groups. Two fecal pellets were collected from each animal via a sterile pipette tip and placed into separate sterile cryovials. Cryovials were immediately placed on dry ice. Gloves were changed between groups. Cryovials were stored at –80°C until shipping on dry ice to lab for analysis. Mice were euthanized by carbon dioxide inhalation at the end of the study, in accordance with the AVMA Guidelines for the Euthanasia of Animals: 2020 Edition. Three mice were euthanized early due to bloat. Pellets were collected in duplicate as a precaution. Some backups were used when DNA extraction of initial samples failed.

### 2.4 Statistical analysis

The microbial community was analyzed using Python version 3.10.12. Richness was calculated as the number of ASVs, species, or genera present in each sample. The non-parametric Wilcoxon rank sum-tests were performed using scipy.stats.ranksums and corrected for multiple comparisons by Bonferroni correction using statsmodels.stats.multitest 0.14.0. Infix edit distance for read filtering was calculated using edlib. Beta diversity based on relative abundance was analyzed at the ASV level using Bray–Curtis dissimilarities obtained from scipy 1.14.1. Groups of samples were deemed “concordant” or “convergent” when their pairwise Bray–Curtis dissimilarities between site average < 0.5 by day 60, a practical threshold previously used to distinguish related vs. disparate airway microbiome samples (Weiser et al., 2022). The among-group differences were determined by the permutational multivariate analysis of variance of the Bray–Curtis distance matrices, implemented in PyPermANOVA (PyPerMANOVA, 2022). For all permutation tests, we used 10,000 permutations. Along with site, we also considered other confounding factors, such as sex and age in the analysis. Principal Coordinate Analyses (PCoAs) were conducted using scikit-bio 0.5.9, which calculates Bray–Curtis dissimilarity values internally based on relative abundance vectors. Visualizations were generated using matplotlib 3.8.0, seaborn 0.13.2, with assistance from calculations using pandas 1.5.3 and numpy 1.26.0. Subregions were extracted using regex 2.5.135.

## 3 Results

### 3.1 Outline of experimental design

**Figure 1:**
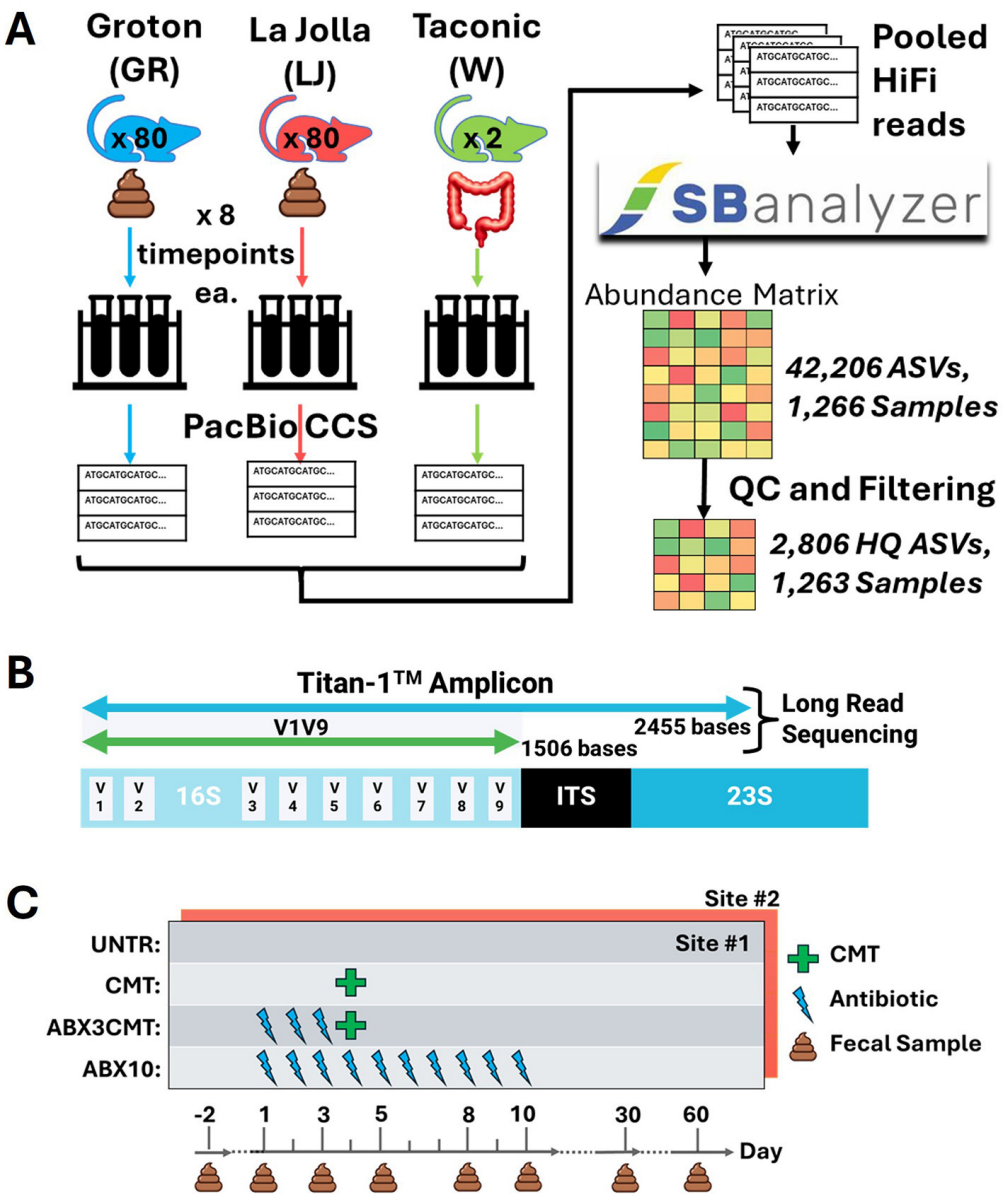
Experimental design **(A)** The fecal microbiomes of 80 mice at each of two laboratory sites (Groton, CT and La Jolla, CA) were sampled eight times over 60 days and sequenced using a high-fidelity long read sequencing method (PacBio CCS). Two replicate samples of cecal material pooled from 15 donor mice are also sequenced and analyzed. All reads were pooled for ASV inference via SBanalyzer and DADA2, yielding 2,806 high quality ASVs across 1,263 samples after filtering. Three mice were euthanized early due to bloat, and three samples were removed due to low read count. **(B)** Sequencing targeted a contiguous region of the rRNA operon about 2,450 bp in length, known as the Titan-1™ amplicon. **(C)** The mice are split into four treatment groups: untreated (UNTR), CMT, antibiotic preconditioning with CMT (ABX3CMT), and ten days of antibiotic treatment (ABX10).

Figure 1 provides a summary of study experimental design, main analysis steps, and observed ASV richness. Briefly—at each of two laboratory sites, Groton and La Jolla, fecal microbiomes of 80 mice were analyzed by sequencing of a contiguous 2,400 base region of the rRNA operon that includes the full 16S, variable length ITS, and partial 23S genes—the Titan-1™ amplicon. Eight timepoints were sampled over 60 days, including the day after the mice arrived on-site (day -2, three days before treatment began).

The Taconic wild-type microbiome (Trubiome^®^) used for CMT was also sequenced, directly from the source vial. Samples were barcoded during PCR and pooled for library construction and sequencing. PacBio CCS (Circular Consensus Sequencing) reads spanning the amplicon were sorted by barcode post-sequencing using SBanalyzer, and ASVs were identified using DADA2 with settings intended to increase sensitivity for low abundance ASVs (Bardenhorst et al., 2022). Quality control and filtering steps were conducted on the resultant matrix, yielding 2,806 high quality ASVs across 1,263 samples, as detailed in the Supplementary Methods.

We analyzed four treatment groups at each site (20 mice per group, 10 per sex): untreated controls (UNTR), cecal microbiome transplant on day 4 (CMT), CMT after 3 days of antibiotic preconditioning (ABX3CMT), and 10 days of antibiotic treatment (ABX10; Figure 1C). Follow-up microbiome samples were taken on days 30 and 60 of the experiment.

### 3.2 Microbiome composition of baseline samples was significantly different between sites

**Figure 2:**
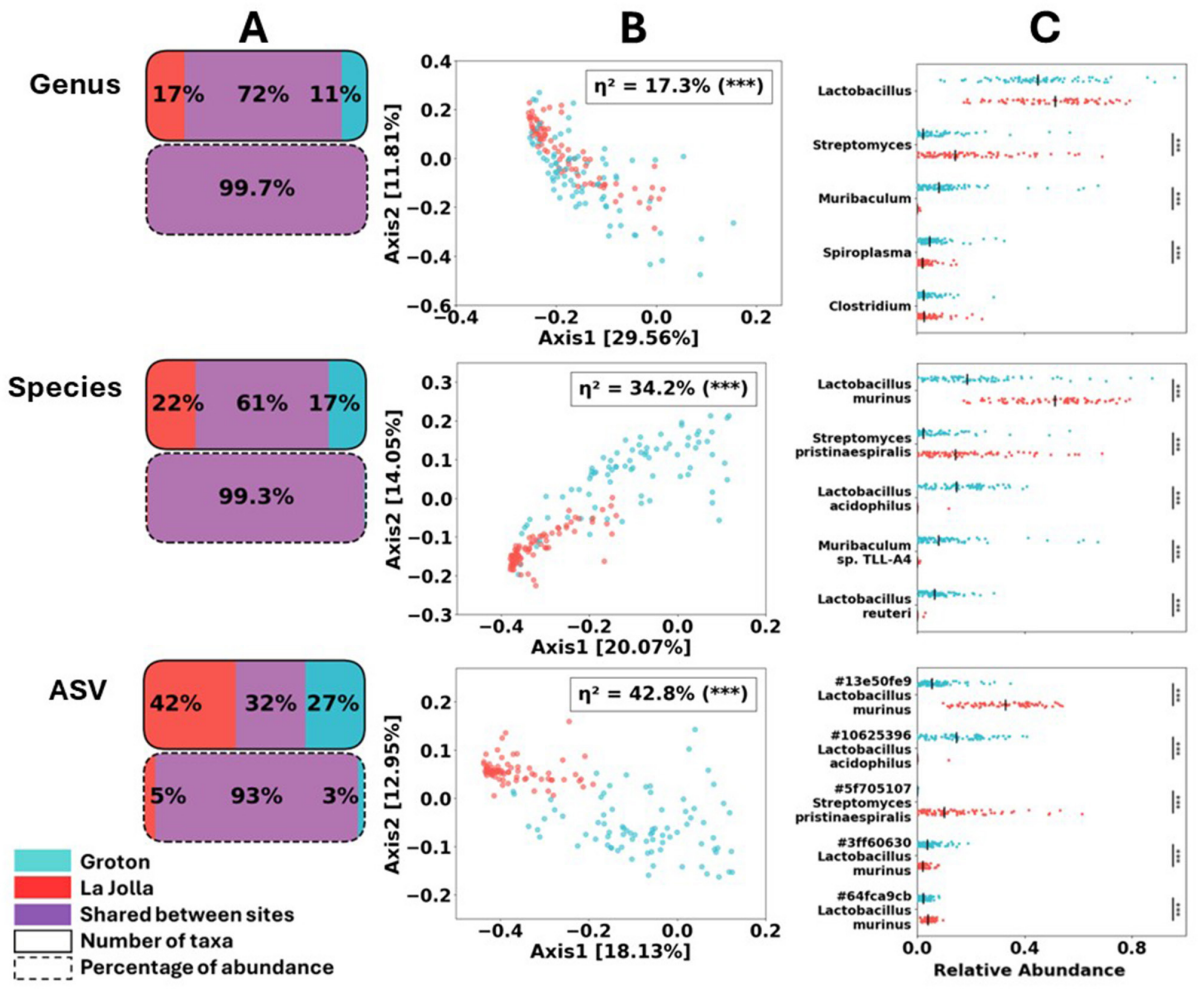
Comparison of baseline (day –2) samples by site. **(A)** Proportion of shared taxa, by both count and relative abundance, decreases radically at ASV-level resolution. Any differences in number of taxa above this level of resolution are due to a few rare taxa. **(B)** PCoA of all samples using Bray–Curtis dissimilarity, computed globally at each level of resolution (only baseline shown) drawn in red for La Jolla, and blue for Groton. η^2^ values obtained via permANOVA, also using Bray–Curtis dissimilarity, confirm most reliable distinction between sites occurs at ASV-level. **(C)** Per-sample variation at each site for baseline samples: relative abundance of the 5 most abundant baseline taxa is depicted along with medians. Significance of difference between sites determined from Wilcoxon rank-sum test.

Gut microbial content at arrival differed significantly between mice shipped to the Groton and La Jolla sites (Figure 2). Trends of taxonomic differentiation, while observable at the genus level (28% non-shared taxa), become more pronounced at the species and ASV level of identification (39 and 68% non-shared taxa, respectively). At the ASV level ≈7.5% of the reads are assigned to non-shared taxa compared to less than 1% at the genus and species levels, suggesting the higher sensitivity of this approach (Figure 2A). Similar trends of unveiling diversity at higher resolution were observed using Principal Coordinate Analysis (PCoA) generated using Bray–Curtis dissimilarity (Figure 2B). The proportion of variance in community composition explained by site (η^2^) from permANOVA tests increases with taxonomic resolution. While sites are significantly differentiated even at the genus level (η^2^ = 17.3%), the magnitude of this difference increases sharply at the species level (34.2%), and is most sensitive (42.8%) when using ASV as our measure of microbial taxon (Figure 2B).

When comparing read counts, with a minimum fold change of 2 and a corrected *p*-value of 0.05 or less, we observe 18 out of 132 genera, 23 out of 254 species, and 94 out of 2,806 ASVs significantly different between the two sites (see Supplementary File S1). Furthermore, when examining the top five most abundant taxa for each level (by read count), we find only three of those five are significantly differentially abundant at the genus level: *Streptomyces, Muribaculum*, and *Spiroplasma* all at *p* < 0.001 using the two-tailed Wilcoxon rank-sum test. At the species level, we see a significant difference across sites at all of those top 5 taxa: *Lactobacillus murinus, Streptomyces Pristinaespiralis, Lactobacillus acidophilus, Muribaculum sp. TLL-A4*, and *Lactobacillus reuteri* are all significant at the *p* < 0.001 level. For the ASVs we also find all top five taxa to be significantly differentially abundant between sites to the *p* < 0.001 level: three *L. murinus* ASVs, one *L. acidophilus* ASV, and one *Streptomyces pristinaespiralis* ASV. See Supplementary File S1 for more comprehensive statistical results. These results showcase the advantages of higher-resolution methods at detailing and contrasting microbial content at species or even ASV level. The rest of our results rely on calculations that are conducted at the ASV level, unless otherwise indicated.

### 3.3 Significant differences persist between sites over 60 days

**Figure 3:**
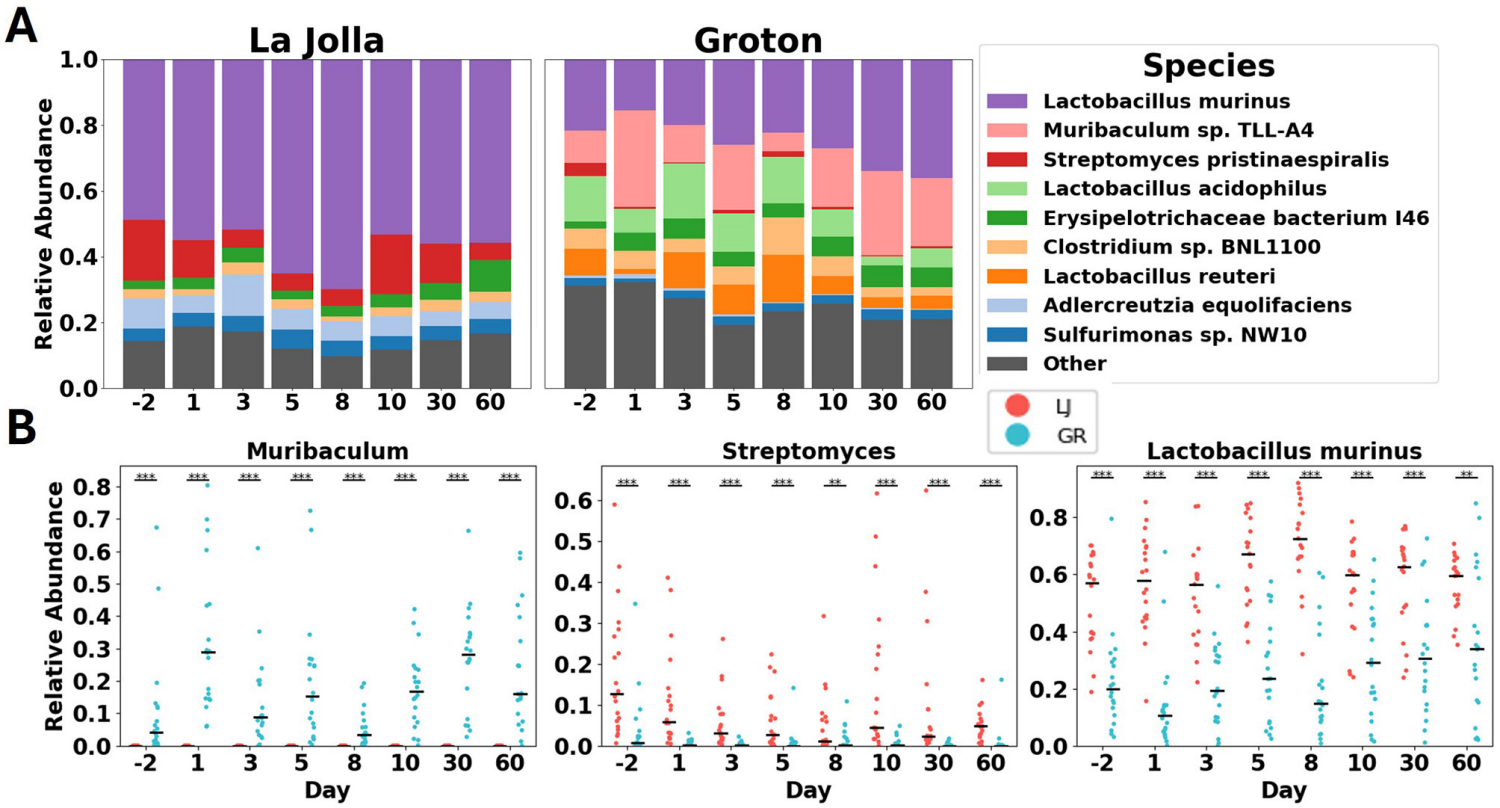
**(A)** shows stacked bar charts of microbiome species' relative abundance in La Jolla and Groton over time. Key species include *Lactobacillus murinus* and *Muribaculum sp. TLL-A4*. **(B)** displays scatter plots for *Muribaculum*, *Streptomyces*, and *Lactobacillus murinus*, comparing abundance over time in La Jolla (red) and Groton (blue). Each panel illustrates significant differences across species and locations.

The differences in microbiome composition observed between control mice from different sites remain significant over the duration of the experiment (Figure 3). While both the species richness and the sets of species detected at the two sites are mostly the same, many of the relative abundances are radically different, and remain so for 60 days (see Supplementary Figure S2). These consistent differences across sites are exemplified in three of the top most abundant bacterial taxa across sites (Figure 3B). *Muribaculum* has consistent abundance, mostly observed in Groton, and remains significantly different between Groton and La Jolla for the duration of the study, at *p* < 0.001 using the Wilcoxon rank-sum test. *Streptomyces* and *Lactobacillus murinus* are found across sites and both are significantly differentially abundant across sites to at least *p* < 0.01 throughout the study, despite some internal variability. *Streptomyces* is detected at a relative abundance of 0.10 ± 0.13 in La Jolla, and 0.01 ± 0.04 in Groton, while *L. murinus* is found at 0.58 ± 0.17 in La Jolla, 0.25 ± 0.20 in Groton. While there is intra-site variability in microbial composition in untreated mice, their microbiomes remain significantly differentiated for the duration of the study to the *p* < 0.001 level using permANOVA with Bray–Curtis dissimilarity (Figure 3; see Supplementary File 2).

### 3.4 Effects of microbial perturbation

**Figure 4:**
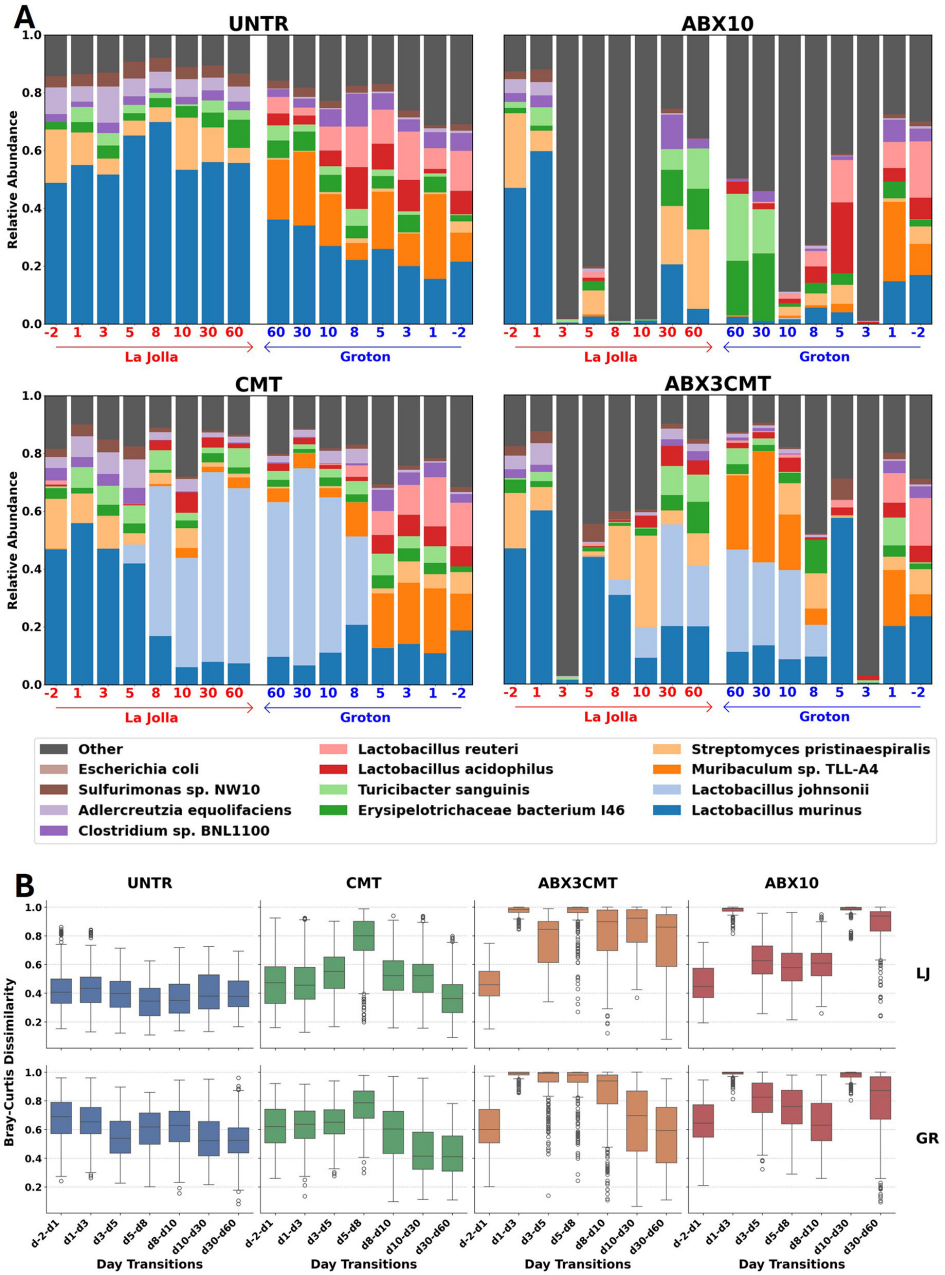
Changes in gut microbiome composition and abundance by treatment group. **(A)** Relative abundances are shown from La Jolla (left) and Groton (right) in each panel for the 12 most abundant species across all samples. The timescale for Groton has been reversed for all treatment groups to enable side-by-side comparison between sites at the conclusion of the experiment. **(B)** Distributions of Bray–Curtis dissimilarities between subjects at consecutive timepoints for each day, site, and condition.

We established treatment conditions to test the short and long-term (1) ability of cecal microbiome transplant to colonize following a single transplant; (2) effects of antibiotic preconditioning on colonization efficacy; (3) re-colonization landscape in the gut microbiome following an antibiotic treatment alone (Figure 4).

While microbial populations in untreated groups remain stable and divergent between sites for the duration of treatment, the microbial perturbations have significant effects on both the within site variance and between site similarity (Figure 4). Strong shifts in major taxonomic groups appear in all conditions, immediately after ABX treatment, first observed changes occurring at day 3 and after CMT introduction, first observed changes on day 8. Following CMT treatment, several ASVs also detected in Taconic are introduced that are not observed on any day in the untreated or ABX10 conditions, or any day prior to day 5. For the ABX3CMT condition 291 ASVs were introduced in Groton and 173 in La Jolla, and for the CMT condition 628 new ASVs are observed in Groton and 617 in La Jolla (see Supplementary File S3). By the last two time points at 30 and 60 days, both sites in the CMT group reached similar stable relative abundances of major taxa (Figure 4). Differences in relative abundance across treatment conditions at the end of the experiment were driven primarily by a few taxa: CMT contained a high relative abundance of *Lactobacillus johnsonii* at both sites (0.61 in La Jolla and 0.54 in Groton), while ABX3CMT contained inconsistent levels of *Muribaculum* sp. TLL-A4 (0.000 in La Jolla, 0.256 in Groton) and *Streptomyces pristinaespiralis* (0.113 in La Jolla, 0.005 in Groton) between sites.

In untreated samples, the Bray–Curtis dissimilarity between consecutive days is relatively stable for the duration of the experiment (Figure 4B). In the CMT group, there is a spike in Bray–Curtis dissimilarity from days 5 to 8. However, the distribution of dissimilarities between days 30 and 60 reverts to levels below those in the untreated samples at both sites (*p* = 0.004 in La Jolla and *p* = 10^−16^ in Groton using a two-tailed *T*-test). That is not the case for either antibiotic condition, where Bray–Curtis dissimilarities between days 30 and 60 continue to exceed untreated dissimilarities from the same site (for ABX3CMT, *p* = 10^−62^ in La Jolla and *p* = 0.008 in Groton, for ABX10 *p* = 10^−71^ in La Jolla and *p* = 10^−56^ in Groton).

Antibiotic treatment resulted in loss of day –2 high-abundance taxa, with low-abundance taxa making up the majority of species detected at day 3 at both sites (Figure 4). In the ABX10 condition during antibiotic treatment, taxa of low-abundance at baseline became dominant. By day 3, the relative abundance of low-abundance species (not in the 12 most abundant designations across all samples) was 0.983 in La Jolla and 0.989 in Groton for ABX10, and 0.971 in La Jolla and 0.972 in Groton for ABX3CMT. This coincided with low alpha diversity during this portion of treatment; an average of 10.8 ± 3.3 ASVs per subject were detected on day 3 in La Jolla and 6.4 ± 2.3 in Groton for the ABX3CMT condition, while an average of 13.3 ± 15.9 ASVs were detected on day 3 in La Jolla, and 6.8 ± 2.6 for ABX10 (Supplementary Figure S2). Following antibiotic discontinuation, stochastic changes in re-colonization resulted in divergent microbial profiles at the two sites. In the ABX3CMT treatment group, the relative abundances of some taxa did settle to similar levels, like *L. johnsonii* with 0.210 in La Jolla and 0.355 in Groton, or *T sanguinis* with 0.095 in La Jolla and 0.055 in Groton. However others are entirely missing from one site by day 60, such as *Muribaculum* in La Jolla (see Supplementary Figure S3 for more information).

**Figure 5:**
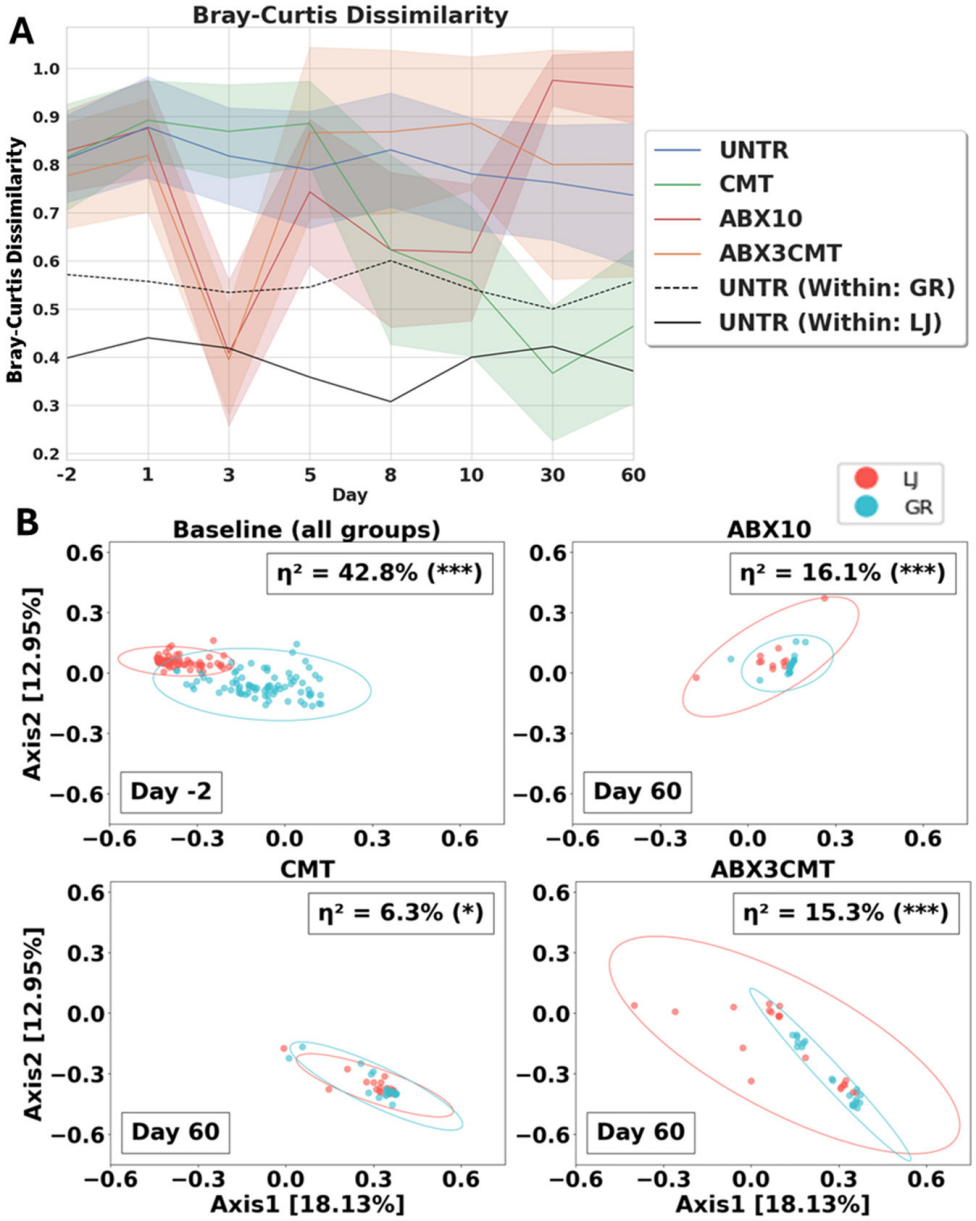
CMT best homogenizes the microbial makeup of mice between sites. **(A)** Bray–Curtis dissimilarity is plotted with standard deviation between sites for all treatment groups, and within site for UNTR as a control. **(B)** Bray–Curtis PCoA of all samples by treatment group, computed globally, only baseline and post-treatment samples shown. Site difference significance determined by permANOVA. Covariance confidence ellipses are drawn to 3 standard deviations.

Cecal microbiome transplant alone leads to convergence of microbial profiles to a within site level of similarity (Figure 5). For CMT-only groups, Bray–Curtis dissimilarity between sites decreased over time (i.e., from 0.816 ± 0.11 to 0.465 ± 0.16, Figure 5A). The changes were robust, directional, and stable, where dissimilarity between sites at the end of the experiment was comparable to the dissimilarity measured within a single site (η^2^ = 6.3%, *p*-value < 0.01 using permANOVA; Figure 5B). ABX3CMT also decreases dissimilarity between sites, with higher variance between the taxa present at the two sites (15.3%, *p*-value < 0.001; Figure 5B). Given the stochastic nature of microbial recolonization following antibiotic treatment, it is possible that antibiotic-treated groups could converge microbiome communities similarly across sites. However, the likelihood of convergence in antibiotic-treated groups would depend heavily on the available antibiotic-resistant strains at each location, whereas convergence observed in the CMT group relies predominantly on the shared inoculum.

To test the robustness of observed convergence at other taxonomic resolutions, we calculated Bray–Curtis dissimilarities at the species and genus levels (see Supplementary Figure S5). Our convergence criterion (mean pairwise Bray–Curtis dissimilarity < 0.5 between site at day 60) was also met at both species and genus levels in the CMT group. However, convergence at genus-level resolution was observed even in the untreated group, indicating that genus-level analyses lack sufficient resolution to detect important differences in microbial community composition observed at ASV-level resolution.

### 3.5 CMT without antibiotic preconditioning is most successful at transferring wild microbes to laboratory mice

**Figure 6:**
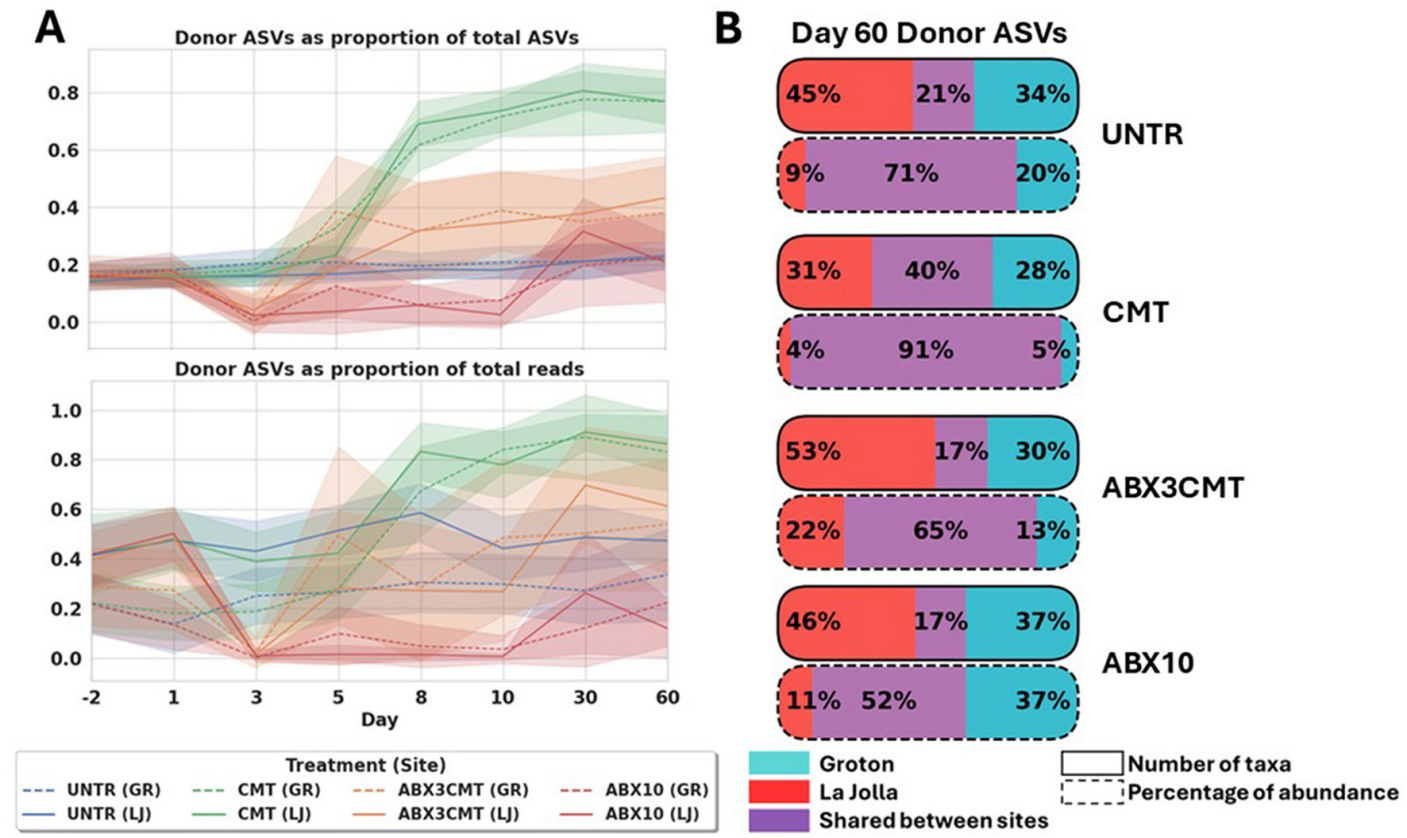
CMT without antibiotic preconditioning is most successful at transferring wild microbes to laboratory mice. **(A)** Relative richness and abundance of donor ASVs over time, by treatment and site. **(B)** Acquisition of donor ASVs at the conclusion of the experiment, by treatment and site.

The number of donor ASVs in the CMT condition at the end of the study is highest of all four conditions (Figure 6). Comparison of total abundance and number of ASVs coming from donor microbes reveals that the ABX3CMT group also experiences an increase in donor ASVs at both sites, with all increases over untreated significant for the ABX3CMT and CMT conditions by day 60 for both sites to at least the *p* < 0.05 level using a *T*-test (all *p*-values less than 0.001 except for the two comparisons in ABX3CMT by proportion of reads—La Jolla at *p*= 0.0383, and *p*= 0.0077 in Groton; Figure 6A). The CMT condition also shows highest colonization efficiency across sites, with 40% of colonized taxa appearing in both sites, as well as 91% of ASV abundance ascribed to donor ASVs belonging to those 40% of shared taxa (Figure 6B). Conversely, the ABX3CMT condition does not surpass conditions that did not receive CMT, in either metric (17% shared taxa and 65% donor ASV abundance stemming from shared taxa).

A specific example of the effective colonization in the CMT condition are the dynamics of the highly abundant *Lactobacillus* genus. *Lactobacillus johnsonii*, a species for which all ASVs are detected in the donor samples, outpaces *Lactobacillus murinus* in abundance at both sites, becoming the dominant species in the genus (Figure 5). In Groton, *L. johnsonii* starts at a relative abundance of 0 ± 0, and goes to 0.54 ± 0.20 in day 60, while the relative abundance of *L. murinus* declines from 0.19 ± 0.16 to 0.09 ± 0.09. In La Jolla, *L. johnsonii* is also not detected on day –2, and rises to 0.61 ± 0.18 by day 60, while *L. murinus* declines from 0.50 ± 0.20 to 0.07 ± 0.08. This occurs for both treatment groups given CMT, but to a much greater extent without antibiotic preconditioning. Within the ABX3CMT condition *L. murinus*, itself having both donor and non-donor ASVs, is detected at lower relative abundances, but not eliminated at either site at the conclusion of the experiment (0.21 ± 0.18 in La Jolla, and 0.11 ± 0.13 in Groton).

**Figure 7:**
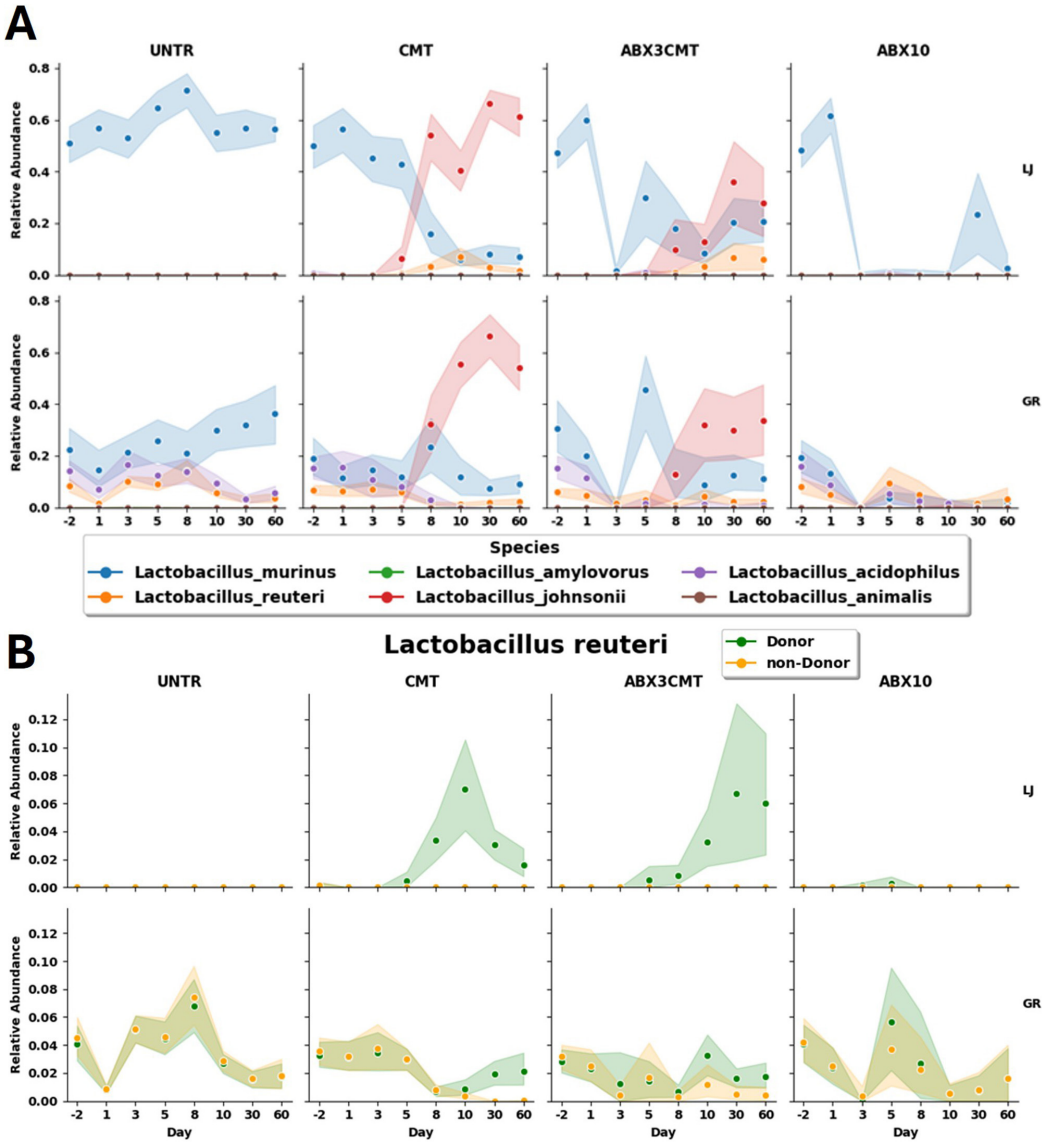
Relative abundances of *Lactobacillus* species shown by treatment and site. **(A)** All *Lactobacillus* species observed, and **(B)** *Lactobacillus reuteri*, subdivided by those ASVs that are detected in donor samples, and those that are not.

A different example reveals a within-species domination of donor ASVs from *Lactobacillus reuteri* (Figure 7B). *L. reuteri* has both donor and non-donor ASVs, both types of which are observed in the baseline microbiome at Groton (with total relative abundances of 0.036 for donor ASVs, 0.039 for non-donor), but neither of which are seen in the La Jolla baseline. Both CMT and ABX3CMT groups experience an introduction of the *L. reuteri* donor ASVs in La Jolla, but only the CMT condition has non-donor *L. reuteri* ASVs reach near-zero levels at Groton. There is also large variance in the relative abundances of donor ASVs for La Jolla in ABX3CMT at 0.06 ± 0.11, indicating inconsistent degrees of donor colonization per-subject when preconditioned with antibiotics.

### 3.6 Antibiotic preconditioning may facilitate the growth of pathogenic bacteria

**Figure 8:**
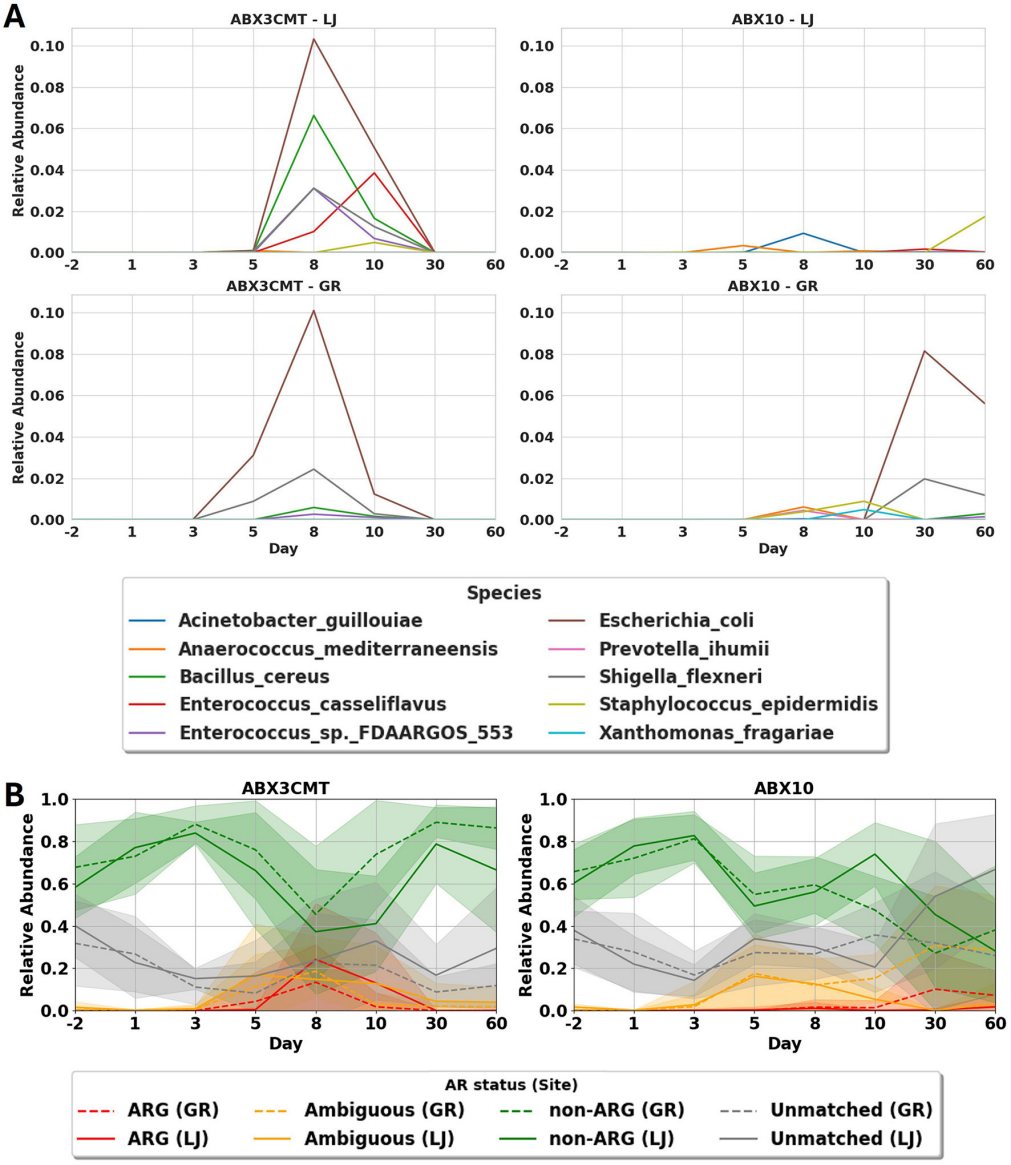
Effects of antibiotic treatment on antibiotic resistant microbial members. **(A)** Mean abundance of the 10 most abundant species assigned to ARG ASVs for antibiotic treated groups, sites combined. See supplemental figure S6 for other conditions. **(B)** Proportion of abundance from the four categories of ASV matches to a database of all complete and partial genomes. This procedure results in four categories of ASVs: those that only occur in genomes with ARGs (ARG ASVs), those that sometimes occur in such genomes (Ambiguous), those that only occur in genomes without ARGs (non-ARG), and those that don't match to any available sequenced genomes at all (Unmatched).

The number of ASVs are temporarily but dramatically suppressed from days 3 to 10 in the ABX10 condition (remaining at 11.9 ± 4.6 in La Jolla, and 14.8 ± 12.0 in Groton by day 10). For the ABX3CMT-treated populations, ASV richness is similarly depressed on day 3 as listed in section 3.4, but has partially recovered by day 10 (53.2 ± 21.3 in La Jolla, and 72.7 ± 30.8 in Groton; Supplementary Figure S2). To understand the landscape of the surviving microbial content in antibiotic treated groups, we identified 45 ASVs that match only to genomes in NCBI containing antibiotic resistant genes (ARGs; see Supplementary Methods). While presence of ARG-positive bacteria associated with pathogenesis is temporary in the ABX3CMT condition, they persist for the duration of study in the ABX10 group, and include *E. coli, Shigella flexneri, Staphylococcus epidermidis*, and *Bacillus cereus*—all of which are known to harm their hosts (Figure 8A). Similarly, for the broader class of ARG related ASVs, they appear temporarily in ABX3CMT group, but are found to persist in ABX10 samples at significant levels in Groton mice (Figure 8B). Three animals in the ABX10 group at La Jolla site developed signs of bloat and were euthanized early. Although the NCBI database is grossly incomplete, and some of these ASVs may appear in unsequenced strains that do not contain ARGs, two species still achieve statistical significance. Within-site relative abundance of *E. coli* and *S. flexneri* increases significantly in the ABX3CMT population at day 8 as compared to baseline using a 1-sample *T*-Test (*p* < 0.001, for both species at both sites), suggesting persistence despite recovering alpha diversity at this time-point (Supplementary Figure S2).

## 4 Discussion

Gut microbiota has been shown to impact immune maturation and recruitment to the gut, yet it is still a commonly uncontrolled factor in many scientific studies in common animal models (Rosshart et al., 2019; Atarashi et al., 2015; Lo et al., 2024). However, despite their experimental advantages, mouse models may not fully recapitulate human microbiota dynamics due to key physiological and microbial ecosystem differences (Nguyen et al., 2015). There are examples of endogenous and exogenous compounds with differential pharmacokinetics driven by bacterial content (Zimmermann et al., 2019a). These effects can be driven by a variety of mechanisms, including alterations of drug structure or bioavailability, as well as poorly understood immune system related effects (Zimmermann et al., 2019b). These findings have gained steadily increasing interest as potential augmentations to treatment options for a variety of indications (Akutko and Stawarski, 2021). Other lines of investigation leverage the opportunity for microbial community to be the standalone treatment option (Cappetto, 2021; Bakken et al., 2011). With mounting evidence about the potential importance of microbial composition in biological responses and metabolic processes for xenobiotics, we aimed to understand the stability and efficiency of controlling microbial gut communities in a nonclinical development setting.

The ability to alter and control the microbial gut content of organisms involves a basic understanding of the composition and dynamics of this community. Deciding how to test the transfer of microbial content, and how to track it, involve a combination of feasibility, cost and genomic resources. In this study, we chose to implement a cecal microbial transfer (CMT) to retain as much of the integrity of the original microbial gut community as possible into the gut of the recipient mice. Additionally, we follow the efficiency of transfer and stability of those bacterial communities in a real-life pharmaceutical development vivarium setting across two, very similarly maintained vivaria, with large sample sizes (*n* = 20/group) and high-resolution, long-read sequencing (PacBio). Long-read sequencing has been demonstrated to provide superior taxonomic and functional resolution in microbiome studies compared to short-read methods, especially shorter amplicons derived from the 16S gene, allowing for improved detection of strain-level microbial dynamics and functional annotation (Gehrig et al., 2022). We demonstrate that, for example, typical V4 16S amplicon genus-level taxonomic assignments are unable to resolve important bacterial sequence shifts that are easily seen using direct ASV-level analysis. Species-level resolution alone can obscure critical strain-level differences that have meaningful biological consequences. For example, ASV-level analysis in Figure 7B revealed that the *Lactobacillus reuteri* strain introduced by CMT entirely displaced the endogenous lab mouse strain, but only in the absence of antibiotics. These particular dynamics are not visible at the genus or species level, and as suggested by Figure 2, obfuscation of similar dynamics may be common at the species level. This highlights the necessity of ASV-level monitoring to generate testable hypotheses regarding bacterial functional differences that are not adequately captured by existing taxonomic classifications. The setup described here allows us to track genomic reference free, sub-strain level metrics to measure and track efficiency of transfer across several methods, impacts of antibiotics and stability of microbial communities in this setting.

Gut microbial composition of nonclinical species is often affected by a multitude of developmental, breeding and sourcing covariates (Ericsson and Franklin, 2021; Ericsson et al., 2015). Specifically, mouse microbiomes are known to vary across vivaria, which could be a result of a host of environmental factors (Hufeldt et al., 2010). Identifying these baseline differences can be a good source for exploring the impact of resolution of microbial identification on the power of community differentiation. Large scale efforts, like the Earth Microbiome Project, focus on unpacking the variation of microbiome on a broad geographic area and at low resolution (Thompson et al., 2017), which provide a fantastic catalog of diversity. In a more constrained environment, where we expect narrower shifts in taxonomic differentiation between individuals, identifying Amplicon Sequence Variants and performing comparisons on that level can identify subtle, but meaningful differences due to treatment (Callahan et al., 2016b). Our findings corroborated the implication that ASV-level analyses are more sensitive than even the highest resolution reference based methods, especially at identifying baseline differences between the microbial gut communities of two sets of mice sourced from different branches of the same vendor.

As microbial composition can impact the findings of animal studies, it is essential to understand the stability of baseline communities in lab animals. While environmental conditions often are a major factor in the alteration of gut communities (Nguyen et al., 2021), lab conditions are highly controlled over time, which led to a rather stable microbial community composition. Each site maintained stable levels of the major taxa identified in the control conditions. Microbiomes at different sites maintained distinct profiles over time, despite identical handling and housing conditions, aligning with findings in human gut microbiomes where individuals tend to harbor relatively stable, distinct microbiome compositions (Arumugam et al., 2011). While levels of overall variation were low, more specific species or ASV-level variants and their fluctuations in laboratory mice have been shown to have significant impacts on experimental outcomes (Mandal et al., 2020), which is why microbial profiling can be a useful tool for tracking temporal microbiome stability as a co-factor for experimental variance.

Introduction of novel microbiota profile in laboratory mice is often recommended in conjunction with antibiotic treatment, as depletion of microbiota can empty currently occupied niches (Keshteli et al., 2017). However, antibiotic pre-treatment can often lead to dysregulation of other functions such as serotonin biosynthesis, intestinal motility (Ge et al., 2017) and metabolic homeostasis (Zarrinpar et al., 2018). Both conditions in our studies that included antibiotic cocktails were successful at temporarily, but drastically down-shifting the community diversity in treated mice. On the other hand, while we see a steeper differentiation between before and after CMT in the antibiotic pre-treated cohort, the dissimilarity metrics in that group remain higher than the CMT only group, suggesting higher variability in the gut microbial communities of CMT treated individuals after antibiotic pre-treatment. Additionally, pre-treatment with antibiotic introduced a higher proportion of bacteria associated with antibiotic resistant genetic content. Although antibiotics introduce uncontrolled variability, in theory, stochastic recolonization after antibiotic treatment could still lead to microbiome convergence. Practically, however, convergence after antibiotic treatment will be strongly influenced by the availability of antibiotic-resistant or commensal bacteria at each independent vivarium. These observations are likely related to a reduction of abundance of sensitive strains and align with the growing body of evidence detailing the potential consequences of antibiotic use on microbial gut health (Patangia et al., 2022).

Treatment with CMT alone was able to consolidate the microbial communities of the two vivaria. Some sources suggest that antibiotic pre-treatment is necessary for such transfer (Keshteli et al., 2017), while others suggest that simple co-habitation is enough for microbial communities to unify (Rosshart et al., 2019; Sun et al., 2023). With stable and different microbiomes in untreated conditions, likely due to sourcing from different vendor sites, we were able to calculate the within site dissimilarity metrics for both GR and LJ vivaria. Comparing the two sites to each other in all treatment conditions reveals that the only treatment that was able to bring the dissimilarity between sites to the levels of within-site dissimilarity was the CMT alone. This suggests that both eliminating the basal microbiome (ABX10) or pre-treating with ABX before CMT both introduce more variability in the resulting communities and don't achieve unification. These results may suggest that the only treatment that successfully and repeatably converged microbiomes to the same final community was CMT alone. Our findings suggest that microbiome standardization across sites could involve only a CMT transfer without further alterations or pre-treatments. However, this study used mice from a single vendor. Additional studies would be required to assess whether cecal microbiome transplantation is effective in mediating convergence of the microbiome composition of mice from different vendors, or whether the same findings hold when using other hosts.

While our study was limited to SPF mice, the findings highlight a broader principle: antibiotic preconditioning, often assumed to “reset” the microbiome, did not produce a stable or consistent baseline. In some cases, it appeared to hinder colonization or promote persistence of undesirable taxa. This raises questions not only for SPF models, but also for wild-type mice and other hosts. These results may have implications for microbiome-based interventions in other mammals, including humans, where antibiotic use could similarly impair establishment of healthy, stable gut microbiota.

In this study we examined potential covariates that impact microbial composition and potentially differential outcomes in nonclinical laboratory settings. We showcase the utility and efficacy of CMT transfer and the superiority of reference free classification of microbiomes in the control and tracking of gut microbial communities in nonclinical studies. These findings can inform strategy for nonclinical study systematization, as well as the understanding of the subtleties of microbial stability and variability in highly controlled lab settings. Mice from one vendor can vary over time and even from a single source, which has been described by us here and others. Differences in mouse gut microbiota populations are an uncontrolled variable that can affect experimental outcomes in meaningful ways. Our findings suggest concrete steps investigators should consider for mouse model studies, including (a) testing prior to any experiment to be sure all animals have consistent, well-characterized microbiota; and if not, (2) using this or similar methods to harmonize all animals prior to running any experiment to ensure reproducible results within and across experiments.

## Data Availability

The original contributions presented in the study are publicly available. This data can be found here: https://www.ncbi.nlm.nih.gov/bioproject/, PRJNA1252082.

## Supplementary material

Supplementary File S1 Differential abundance testing results between Groton and La Jolla at ASV, species, and genus levels. Contains tables detailing Wilcoxon rank-sum tests, raw and corrected p-values, significance levels, and log-fold changes of taxa abundances between sites on day -2. Supports the statistical results reported in Figure 2.

Supplementary File S2 PERMANOVA statistical comparisons between Groton and La Jolla across time points and treatment groups. Provides exact permANOVA statistics including p-values, eta-squared effect sizes, and F-statistics used to quantify microbiome differences over time. Supports results in Figures 2, 3, 5.

Supplementary File S3 List of newly introduced ASVs detected after CMT treatments at each site. Contains details for each introduced ASV including site, treatment group, ASV ID, day of first appearance, relative abundance at appearance, genus/species taxonomic assignment, and full taxonomic classification. Supports analysis shown in Figure 4.

Supplementary Figure S1 Relative abundance of phyla over the course of the treatment.

Supplementary Figure S2 Alpha diversity at each timepoint.

Supplementary Figure S3 Per-sample relative abundances of Muribaculum for the four treatment groups at both sites.

Supplementary Figure S4 Factor analysis for **(A)** site and **(B)** sex using permANOVA with Bray–Curtis dissimilarity. Heatmap depicts p-values, superimposed circles depict η^2^.

Supplementary Figure S5 Bray–Curtis dissimilarity between site is calculated at species and genus resolution.

Supplementary Figure S6 Proportion of samples attributable to the four types of ARG ASV genome matches.

Supplementary Figure S7 Relative abundance values of microbiota for which the mice were SPF.

Supplementary Figure S8 Proportion of taxa shared between site at baseline, by both count and relative abundance, as would be identified based on subregions of the 16S gene (extracted from ASVs using regex pattern matching of common degenerate primer sequences).

Supplementary Figure S9 PCoA of all samples, computed globally at each level of resolution (only baseline shown) drawn in red for La Jolla, and blue for Groton.

Supplementary Figure S10 Per-sample variation at each site for baseline samples using different subregions: relative abundance of the 5 most abundant baseline taxa is depicted along with medians.

Supplementary Figure S11 Initial Filtering. (A) Distribution of infix edit distances for reads to assigned ASVs. (B) Read retention for various thresholds on infix edit distance of read to assigned ASV.

Supplementary Figure S12 Initial filtering.

Supplementary Figure S13 Per-ASV abundance vs prevalence in samples.

Supplementary Figure S14 Filtering of ASVs near to highly abundant ASVs.

Supplementary Figure S15 Distribution of sample depth by treatment group.
