# Supplementary material for: Cecal microbiome transplantation without antibiotic preconditioning standardizes murine microbiomes
Source: Front Microbiol. 2025 Aug 11;16:1632210. doi: 10.3389/fmicb.2025.1632210 (PMC12376172; doi:10.3389/fmicb.2025.1632210)
Supplement: Supplementary Figure S1 — Relative abundance of phyla over the course of the treatment. [file Data_Sheet_4.pdf]

Supplementary Material

SUPPLEMENTAL FIGURES

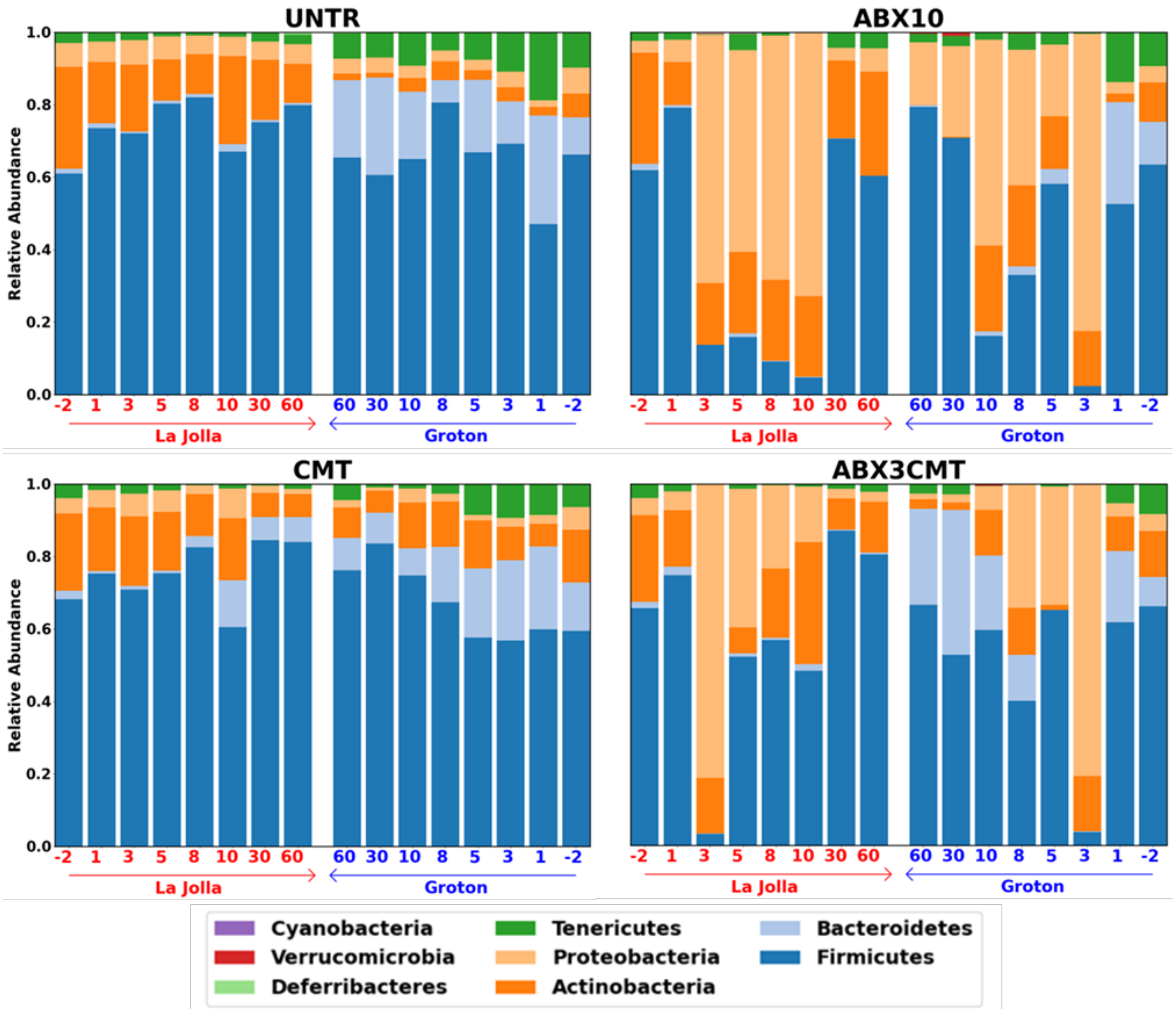

Figure S1: Relative abundance of phyla over the course of the treatment.

The timescale for Groton has been reversed to show convergence at the conclusion of the experiment.

Analysis of ASV counts revealed that by day 3, antibiotic treatment dramatically reduced alpha diversity in both ABX groups, with gradual recovery observed following cessation of treatment. In contrast, the CMT group maintained stable alpha diversity throughout treatment, showing a slight increase by day 10 (Fig. S2).

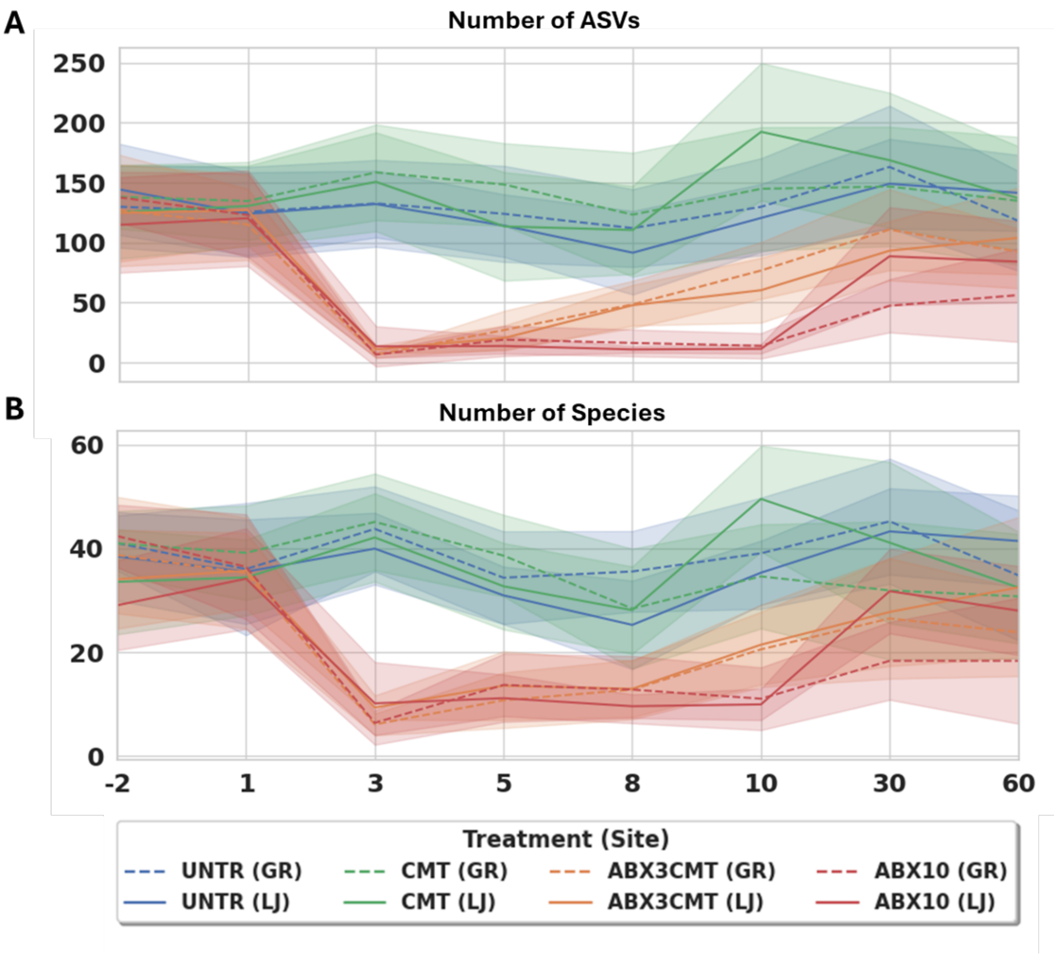

Figure S2: Alpha diversity at each timepoint

Average and standard deviation of number of (A) ASVs and (B) species detected per subject at each timepoint.

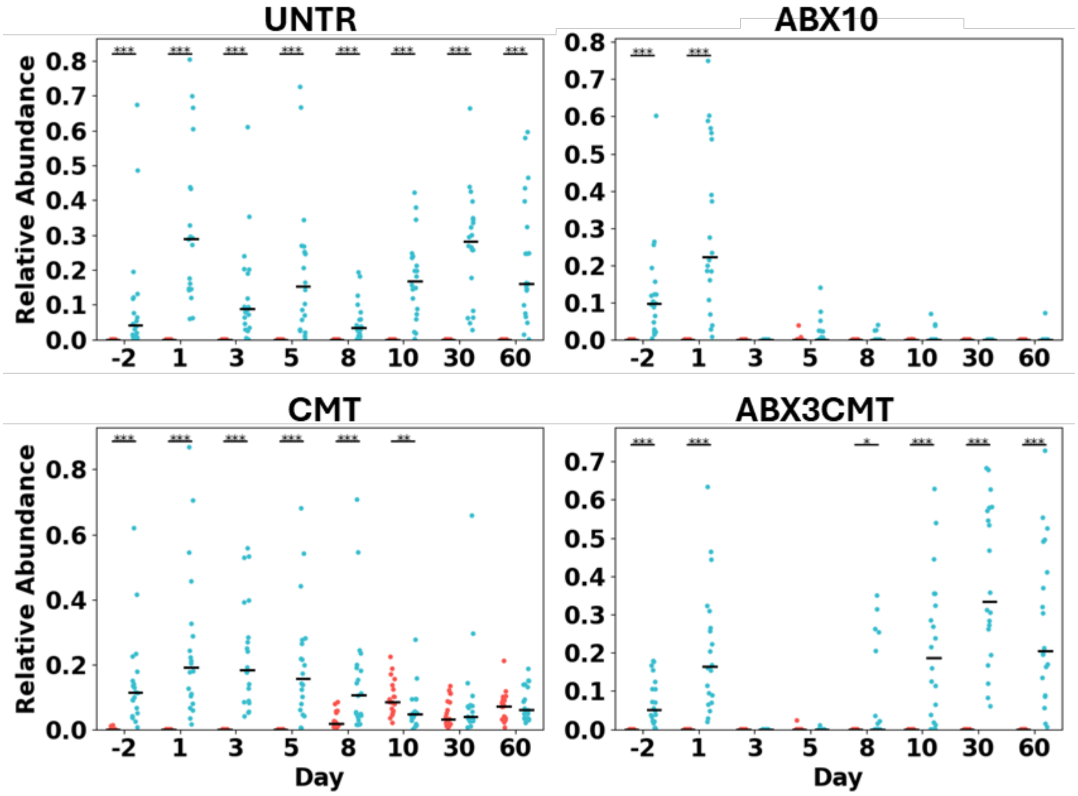

Figure S3: Per-sample relative abundances of Muribaculum for the four treatment groups at both sites.

Median plotted as small black bar. Significance tests are conducted between sites with the two-tailed Wilcoxon rank-sum test.

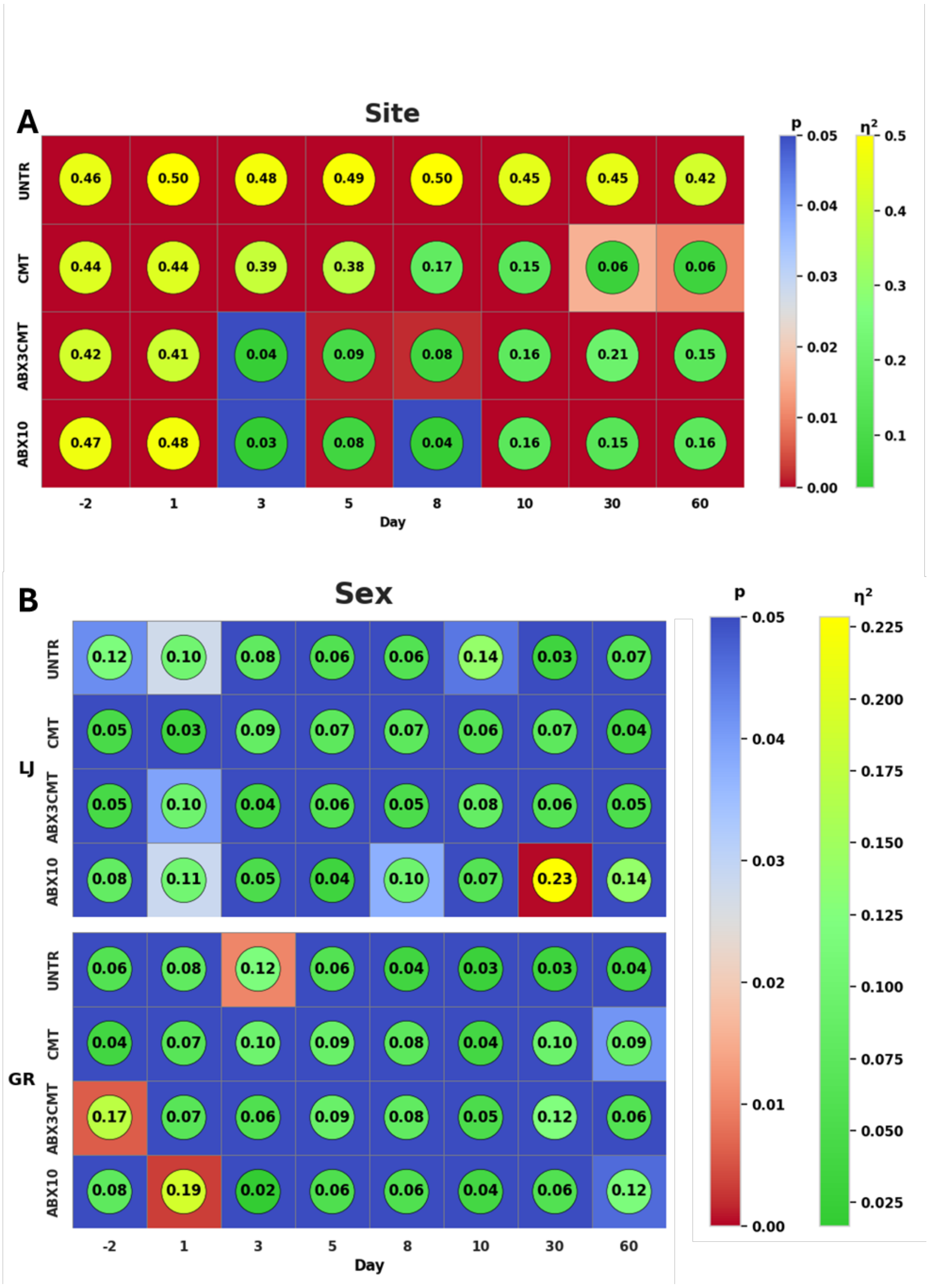

Figure S4: Factor analysis for (A) site and (B) sex using permANOVA with Bray-Curtis dissimilarity. Heatmap depicts p-values, superimposed circles depict η^2.

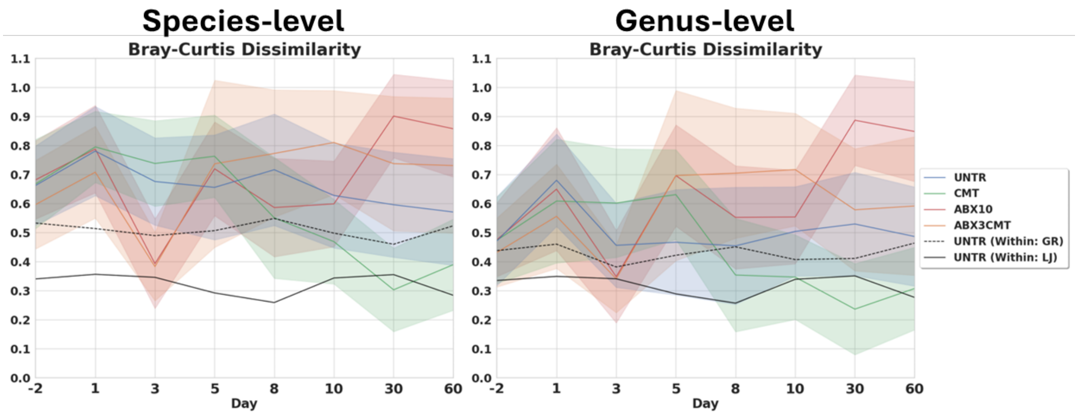

Figure S5: Bray-Curtis dissimilarity between site is calculated at species and genus resolution.

Mean values are plotted with standard deviation for all treatment groups, and within site for UNTR as a control.

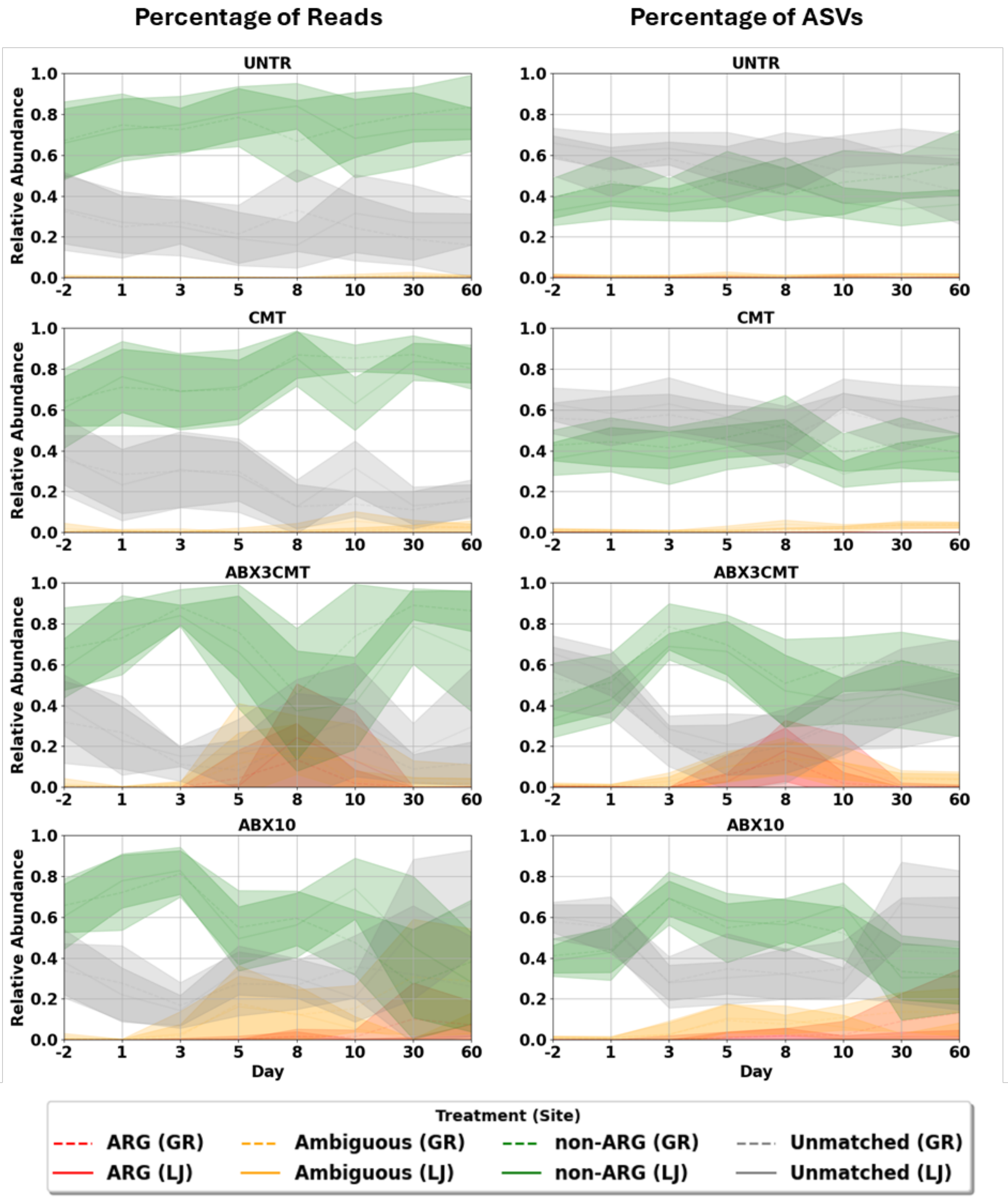

Figure S6: Proportion of samples attributable to the four types of ARG ASV genome matches.

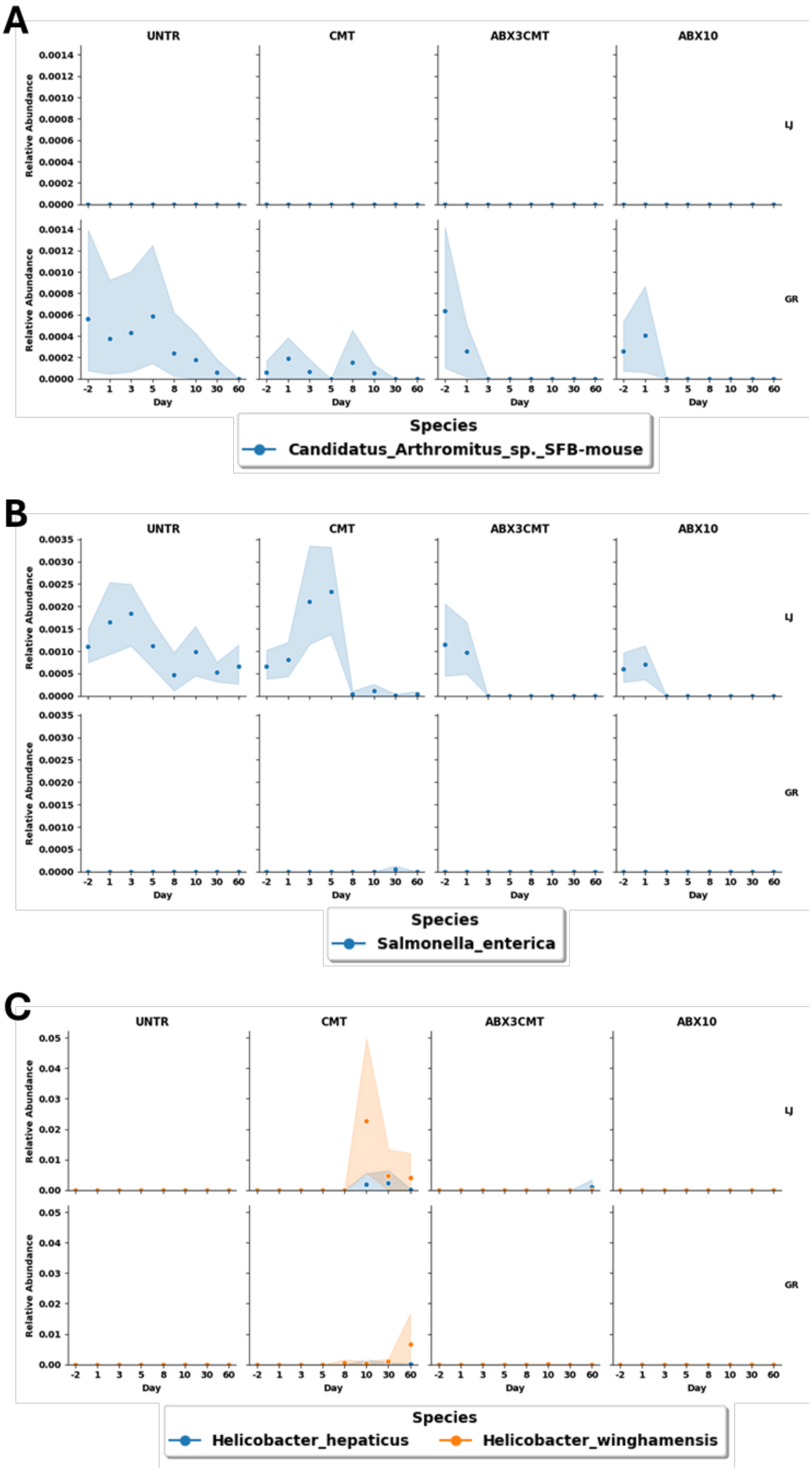

Figure S7: Relative abundance values of microbiota for which the mice were SPF.

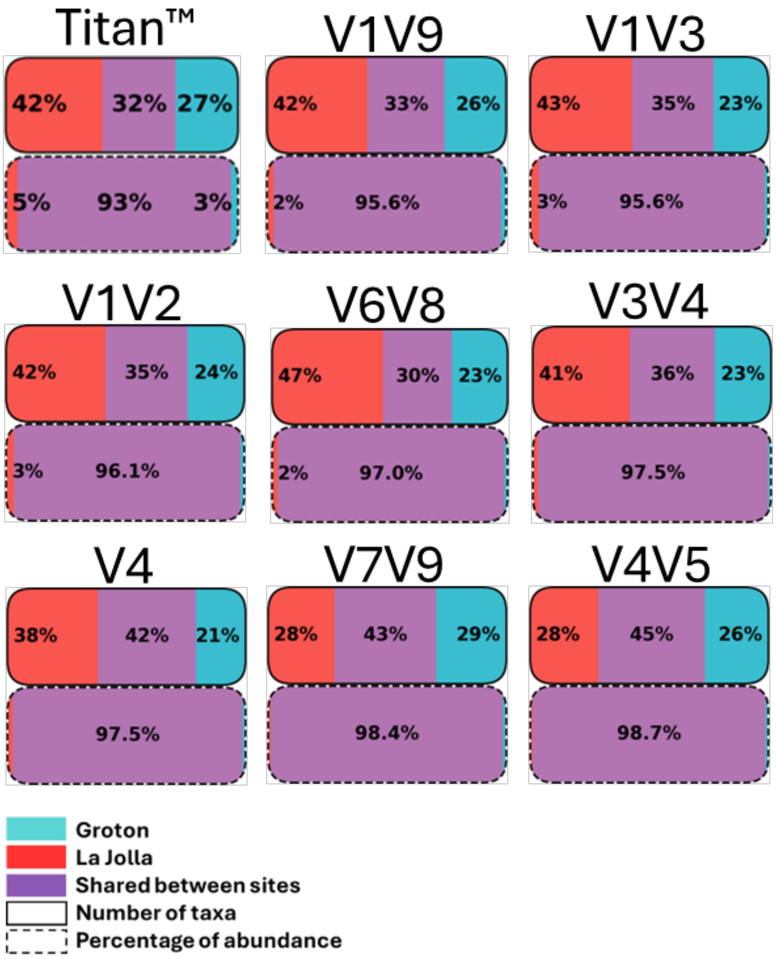

Figure S8: Proportion of taxa shared between site at baseline, by both count and relative abundance, as would be identified based on subregions of the 16S gene (extracted from ASVs using regex pattern matching of common degenerate primer sequences).

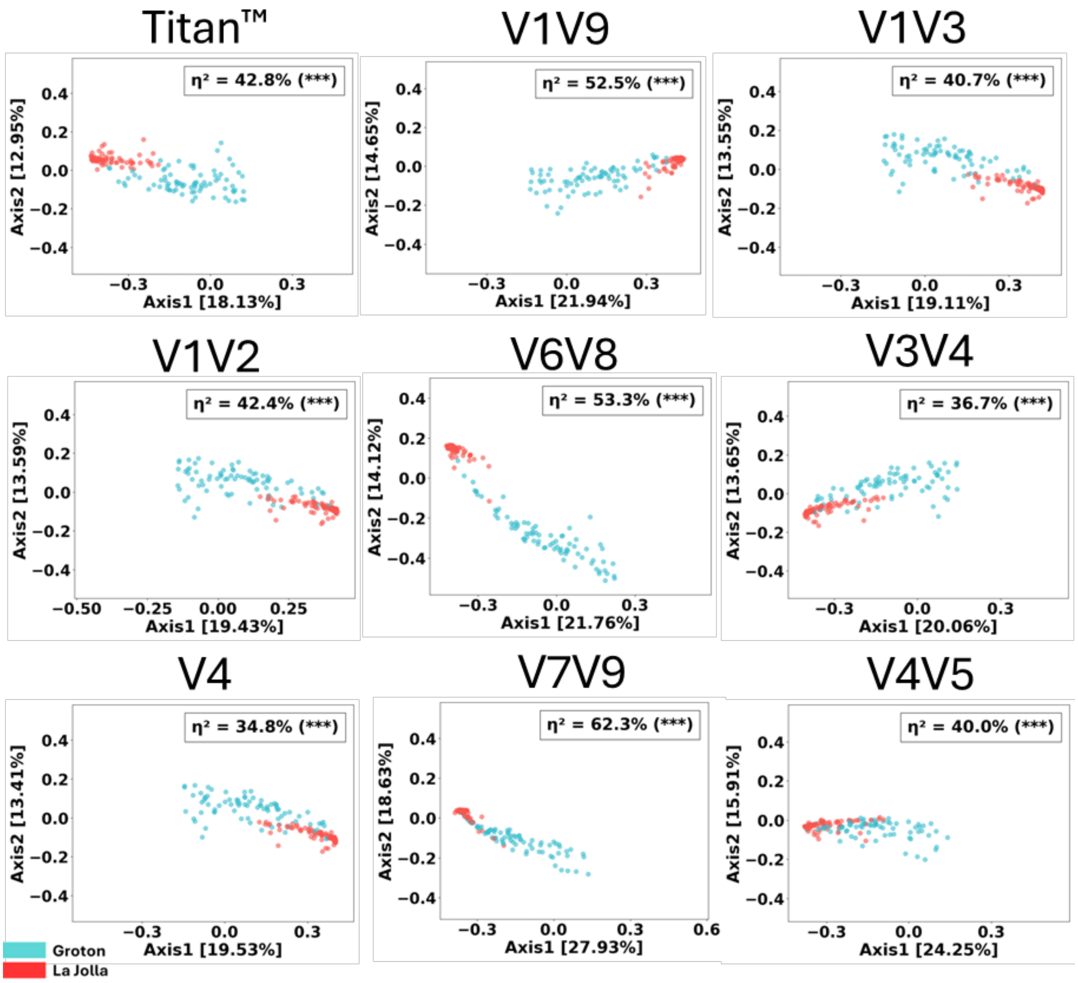

Figure S9: PCoA of all samples, computed globally at each level of resolution (only baseline shown) drawn in red for La Jolla, and blue for Groton. η^2 values obtained via perMANOVA are depicted along with significance level. Resolution levels are subregions of the 16S gene extracted from ASVs using regex pattern matching of common degenerate primer sequences.

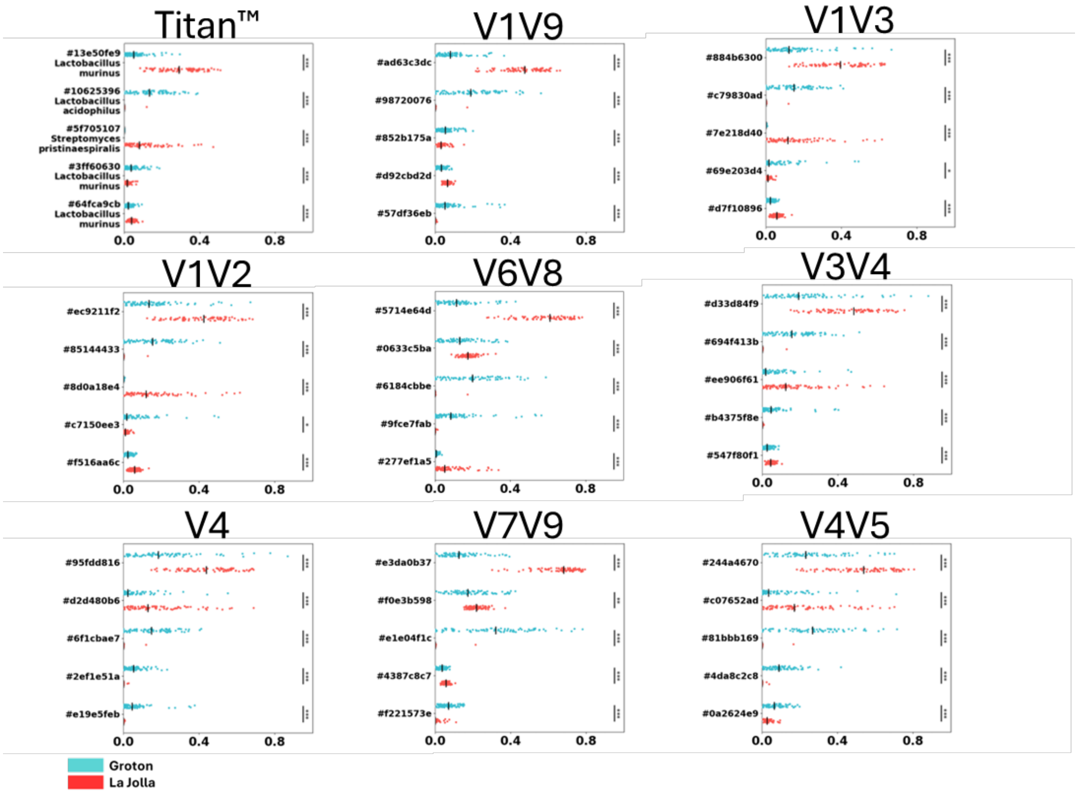

Figure S10: Per-sample variation at each site for baseline samples using different subregions: relative abundance of the 5 most abundant baseline taxa is depicted along with medians. Significance of difference between sites determined from Wilcoxon rank-sum test. Resolution levels are subregions of the 16S gene extracted from ASVs using regex pattern matching of common degenerate primer sequences. Long-read sequencing (Titan-1™, V1-V9) has been shown to improve taxonomic resolution compared to short-read approaches [23].

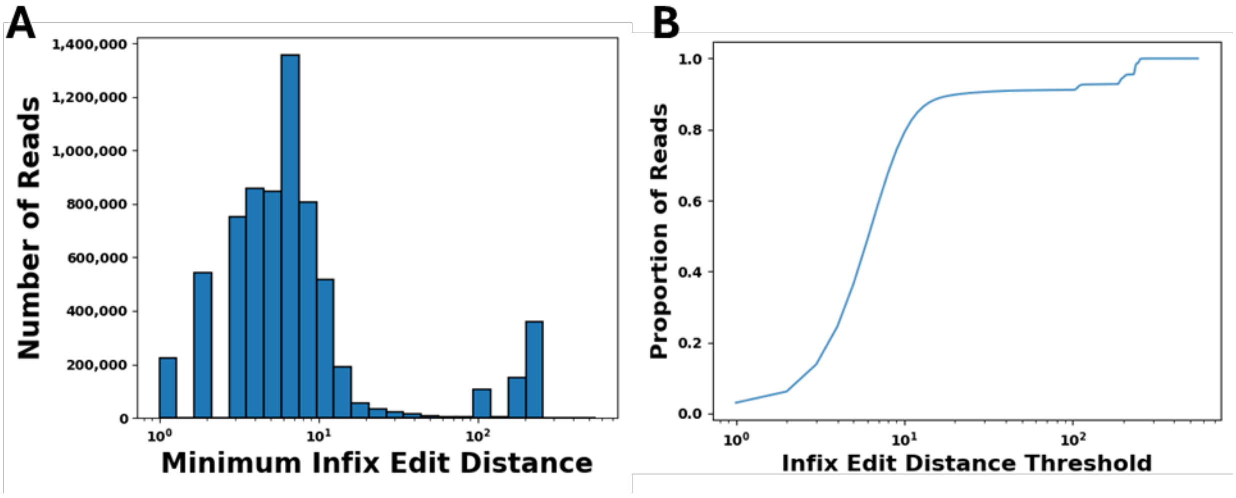

Figure S11: Initial Filtering. (A) Distribution of infix edit distances for reads to assigned ASVs. (B) Read retention for various thresholds on infix edit distance of read to assigned ASV.

SUPPLEMENTAL METHODS

Sequencing Data Processing

Several limitations prohibited analysis of all sequencing data simultaneously. However, to heighten the likelihood of identifying rarer taxa, the pipeline was supplied with a maximal number of sequences from which to infer ASVs. Several sequencing runs were conducted. Each run was individually demultiplexed and classified by mapping using SBAnalyzer. Any read without an exact double-sided barcode match was excluded to maximize confidence in sample assignments. After demultiplexing, the sample assignment for each was encoded in the FASTQ. All such reads were aggregated into subsets by their classified genus. ASV inference and error correction was performed using DADA2 on these genus subsets, as DADA2's run time and memory requirements increase quadratically with the number of unique input sequences. Two genera exceed 1M reads, Lactobacillus and ”Unclassified”, and these were further divided into eight subsets for ASV inference and error correction. The ASV assignment of each read was tracked for later comparison. The resulting ASV frequency tables were merged.

After aggregation, we note large infix edit distances from some reads to their assigned ASV.

The bulk of reads were within about 30 BPs of their ASV. By requiring that reads be at most 30 BP from their assigned ASVs, about 90% of the reads were retained. Further analysis of the reads that do not meet this criterion may prove beneficial for future analyses but will be excluded here.

After filtering low-fidelity reads, the data still showed many low-abundance ASVs. As recommended by the authors of the pipeline [10], a simple filter was employed: exclude any ASV that has fewer than 10 reads. We also chose to exclude any ASV detected in only one sample from further analysis.

Several spurious ASVs remained after these filtering steps. For each highly abundant ASV, many low-abundance ASVs occurred near to it in sequence space, usually just one base pair away. To mitigate this phenomenon, ASVs are excluded if they are 1 BP from any ASV 1000 times as abundant or more.

Some samples were of very low abundance. The samples treated with antibiotics cannot be filtered by abundance, as the treatment itself decreased alpha diversity to near zero. However, the abundances of the

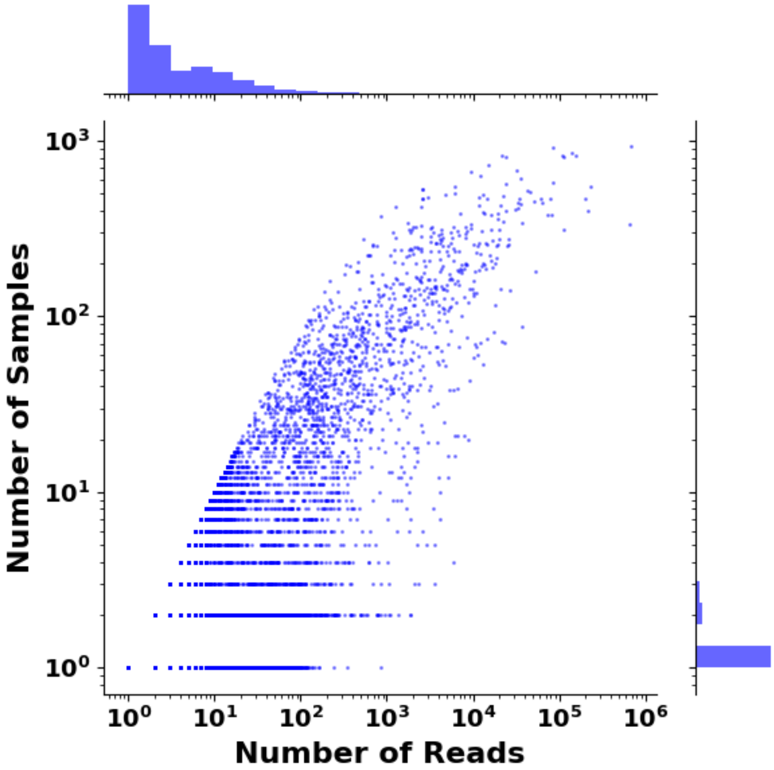

Figure S12: Per-ASV abundance vs prevalence in samples

untreated and CMT samples appeared to follow a normal distribution. Any sample with fewer than 500 reads was excluded from further analysis.

ARG Analysis

To identify antibiotic-resistant organisms in this dataset, all 337,029 complete and partial genomes accessible from NCBI Refseq Genome Database on 02/20/2024 were downloaded, and a BLAST DB was created. All antibiotic-resistant gene (ARG) sequences from resFinder and CARD were also downloaded, and every sequence whose presence indicates antibacterial resistance of any kind was searched against this DB. We adopted a threshold used previously, in projects like the Human Microbiome Project, to identify homology in BLAST results for similar searches: at least 90% coverage, and at least 95% nucleotide identity. If any ARG matches to a region in a genome, we consider that genome to be of an antibiotic resistant organism. Further information exists for some sequences on the specific antibiotics to which the organism is susceptible, but as the mice in this experiment were given several broad-spectrum antibiotics, it is sufficient to test for antibiotic resistance of any kind.

We also extracted all Titan-1™ regions from the genomes downloaded, yielding 428,397 operon regions, representing 184,493 distinct sequences. An ASV from the mouse gut data was considered a match to a Titan-1™ region extracted from these genomes if global alignment yielded at least 98% identity.

Finally, an ASV is then considered yielded from an antibiotic resistant organism if it matches only to genomes that in turn contain matches to at least one ARG. That is, an ASV is antibiotic resistant if it is found exclusively in antibiotic resistant genomes. As there is much sharing of rRNA operon sequences

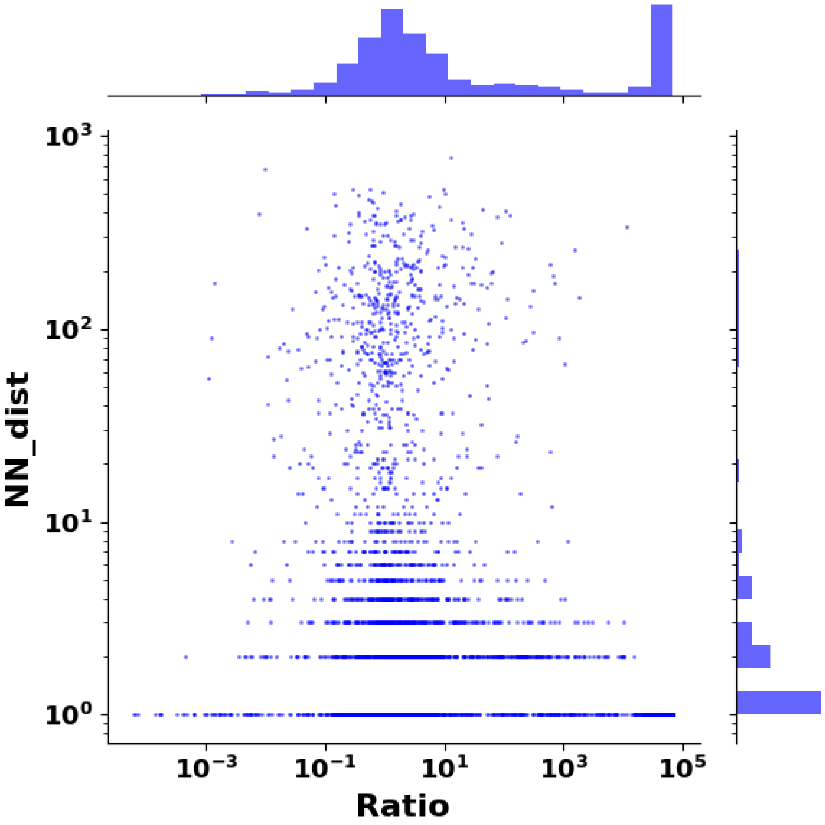

Figure S13: Filtering of ASVs near to highly abundant ASVs.

between strains of a species, this is necessary to ensure a minimum of false positives. We readily admit this procedure allows for many false negatives for the ambiguously antibiotic resistant ASVs.

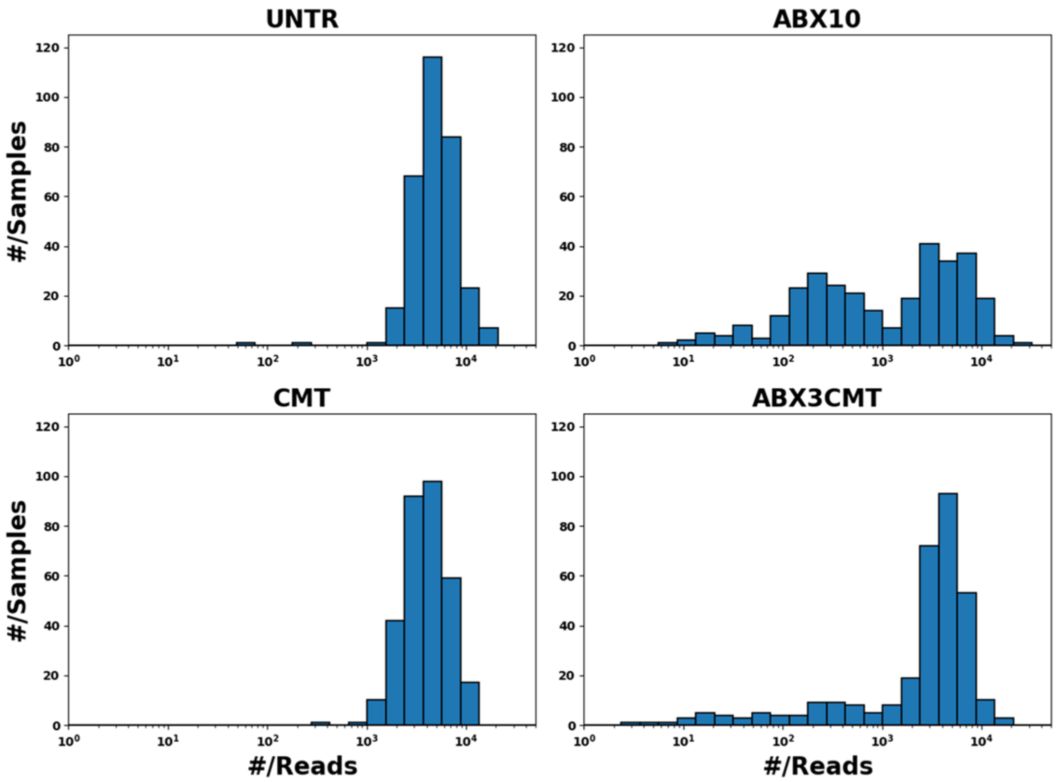

Figure S14: Distribution of sample depth by treatment group.

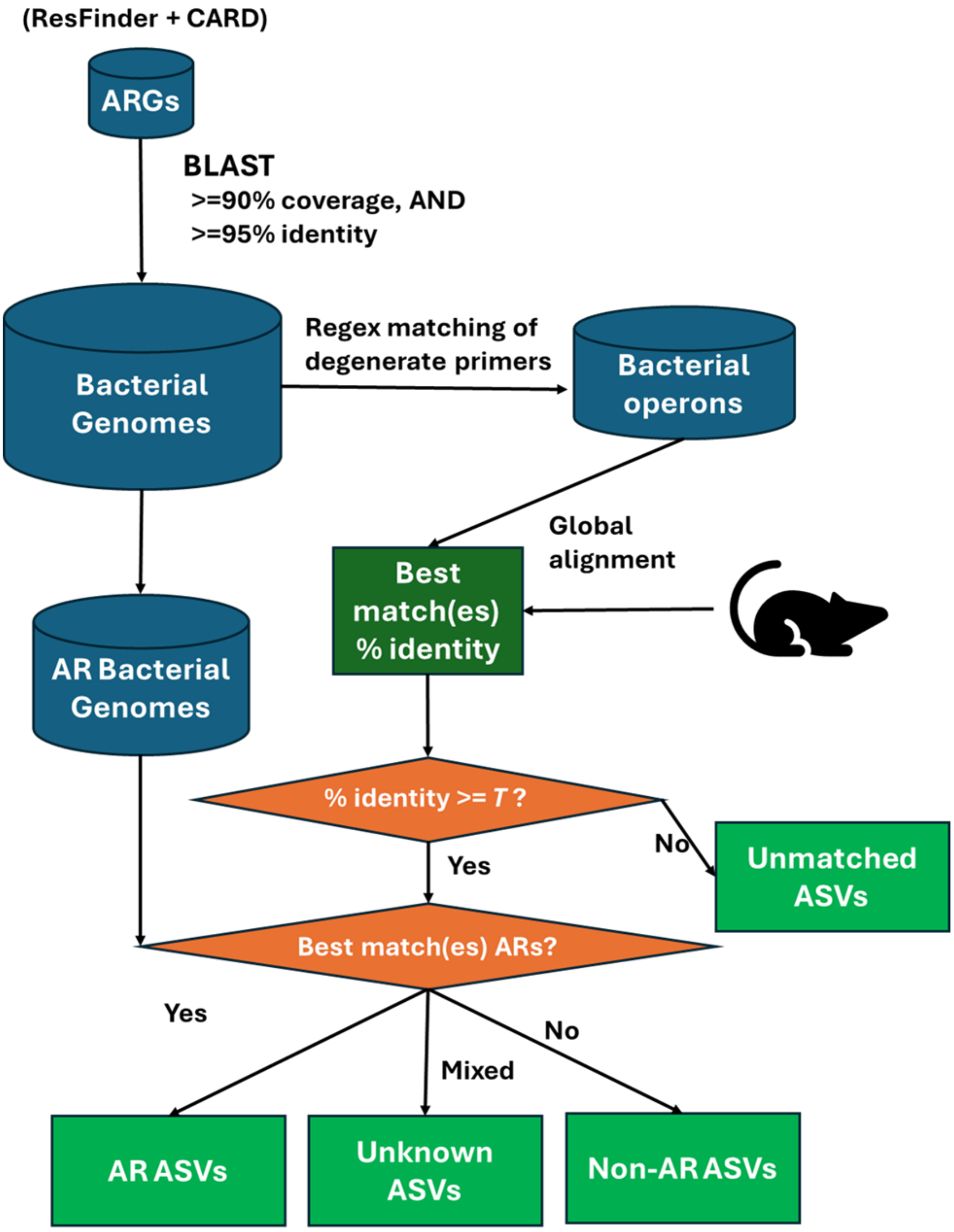

Figure S15: Matching experimental ASVs to antibiotic-resistant organisms
